# Principles Entailed by Complexity, Crucial Events, and Multifractal Dimensionality

**DOI:** 10.3390/e27030241

**Published:** 2025-02-26

**Authors:** Bruce J. West, Senthil Mudaliar

**Affiliations:** 1 Center for Nonlinear Science, University of North Texas, Denton, TX 76203, USA; 2 Department for Research and Innovation, North Carolina State University, Raleigh, NC 27606, USA; 3 Uniformed Services University of the Health Sciences, Bethesda, MD 20817, USA; senthil.mudaliar@usuhs.edu

**Keywords:** complexity synchronization (CS), crucial event (CEs), multifractal dimensionality, empirical principles, fractal architecture (FA)

## Abstract

Complexity is one of those descriptive terms adopted in science that we think we understand until it comes time to form a coherent definition upon which everyone can agree. Suddenly, we are awash in conditions that qualify this or that situation, much like we were in the middle of the last century when it came time to determine the solutions to differential equations that were not linear. Consequently, this tutorial is not an essay on the mathematics of complexity nor is it a rigorous review of the recent growth spurt of complexity science, but is rather an exploration of how physiologic time series (PTS) in the life sciences that have eluded traditional mathematical modeling become less mysterious when certain historical assumptions are discarded and so-called ordinary statistical events in PTS are replaced with crucial events (CEs) using mutifractal dimensionality as the working measure of complexity. The empirical datasets considered include respiration, electrocardiograms (ECGs), and electroencephalograms (EEGs), and as different as these time series appear from one another when recorded, they are in fact shown to be in synchrony when properly processed using the technique of modified diffusion entropy analysis (MDEA). This processing reveals a new synchronization mechanism among the time series which simultaneously measures their complexity by means of the multifractal dimension of each time series and are shown to track one another across time. These results reveal a set of priciples that capture the manner in which information is exchanged among physiologic organ networks.

## 1. Introduction

This tutorial is intended to satisfy an *unmet need in medicine regarding how to extract information on real-time inter-organ communication from physiological time series (PTS)*. Unfortunately, a relatively common occurrence in clinical medicine is a patient that “looks well” and then suddenly and rapidly clinically declines. There is thus a pressing need to revisit how we analyze a patient’s PTS to ensure that we are not missing any information that could help improve bedside management and ultimately patient outcomes. The source of a PTS is the nested multiscale anatomical structure of the human body: intra-cellular communication networks are nested within cellular communication networks, which are contained within organs, which give rise to communication networks between organs, and so on, ultimately giving rise to the human body. Moreover, there exists vertical communication between each of these hierarchical layers.

The ideal signal processing paradigm for a PTS must explicitly capture information flow among the human body’s nested networks. Moreover, given that the material realities of information processing are likely distinct among the body’s nested network—for instance, even though the heart, brain, and lungs (HBL-triad) communicate with each other, cardiac cells behave differently than repiatory cells, and each of those cells behaves differently than the cells that comprise the brain—there must be a way that the body nonetheless is able to coordinate these materially different organ networks (ONs).

The simplest hypothesis is that information flow among the materially diverse physical networks that comprise the human body occurs in accordance with a shared primary language that can be understood *across all scales*. This primary language must have a preserved mathematical structure that governs information flow. Logically following through on this hypothesis ultimately reveals—from substantial empirical investigations—that the mathematical structure of this information flow is fundamentally fractal [1,2]. A key result in support of the universal role of fractals in the body is that PTS corresponding to the HBL-triad have very distinct signal morphology, but their respective fractal properties converge to a shared signal morphology [1,2].

Consequentially, we establish in this tutorial the following signal processing paradigm for PTS:

o Assume that all PTS are fractal unless signal analysis explicitly proves otherwise.
o This entails an assumption that all PTS are generated by non-Gaussian statistical processes and are themselves not Gaussian.

### 1.1. Principles of Organ Network Communications

Scientific principles are at work at the fundamental level of the scientific method, such as formulating a hypothesis, designing an experiment to test the hypothesis, and collecting datasets which can be interpreted to either support or reject the hypothesis. Principles differ from laws in that the latter describes events but do not explain why the events happen, whereas the former tells us the why and the how of the things that do happen. For example, *Heisenberg’s Uncertainty Principle* addresses the lack of certainty encountered in simultaneously measuring canonical pairs of physical variables. Consequently, measuring the location of an event to an uncertainty Δx entails an uncertainty Δp of being able to measure the simultaneous canonical momentum such that its product is greater than a given constant. The principle is therefore a qualitative statement cocerning the quantitative nature of physical measurements. In the example given when the position of a microscopic object, say an electron, is known with certainty ΔX=0, the uncertinty in its momentum is $\Delta P_{x}=\infty$ and consequently cannot be known at all. In addition, more particular principles are characteristic of specific scientific disciplines and influence the methods of observation; the acquisition, storage, management, and sharing of data; and the communication of scientific knowledge and information.

This tutorial explores the various ways complex networks in the life sciences, referred to herein as organ networks (ONs), share information with each other. The key word here is complexity in all its many guises and the information we quantify with these various forms. Researchers with backgrounds in science, technology, engineering, and mathematics (STEM), in short, from virtually every research discipline have focused on complexity and its understanding as being the conceptual barrier to the understanding of their discipline in a modern world context. The term barrier is used advisedly because complexity is ill-defined, as was made apparent in the formation of the *Complexity Science Hub Vienna* [3] in which every founding member of this ambitious international collective had a different vision of complexity—“43 visions” to be precise. This is not meant as criticism but merely as one indication of the general difficulty of articulating the dynamics of complexty and its relation to crucial events (CEs).

Herein, we focus on the different ways crucial events (CEs) enable us to think about complex phenomena primarily in the life sciences, although much of what is documented in medicine can also be found in other disciplines as well. In an earlier colloquium, we [4] explored the more obvious reasons why traditional statistical processes, including those described by *integer-order probability calculus* (IOPC), are not sufficient to capture the full range of dynamics found in natural and man-made processes and events. Specifically, the complexity of nonlinear dynamic processes demand that we extend our functional horizons beyond the analytic and into analyses suggesting that the functions of interest lack traditional equations of motion [5]. To explore this lack of traditional support, we introduce fractal architecture (FA) into the ways we consider how Nature overcomes adversity to regain healthy function, particularly through the use of fractal structures, fractal statistics, and fractal dynamics. The method we use is to never to impose fractality on a model without an obvious empirical invitation but when it is found in the data to determine which of the many fractal functionalities explains its presence and only then to develop a workable and verifiable fractal model of the structure, the dynamics, or the statistical behavior of the phenomenon under study.

A recurring theme of interest herein is the importance of operational time in medical science as emphasized by Buzsáki in his remarkable book [6] in which he described how the device of the human brain utilizes information gathered from its environment to attach the notion of ‘duration’ to the time sequencing of the occurrence of CEs. lt becomes clear as the narrative proceeds why ‘time’ is used as the quantity to clarify how the brain operates and what that entails regarding the dominance of information transfer over that of energy transfer in the normal operation of the mammalian brain.

The prequel to the present talk [4] concentrates on fractional-order calculus (FOC) as a way of addressing the inherent dynamic complexity of such common physical phenomena as turbulence [7], the neurological activity of brainquakes [8], and habituation [9]; familiar social phenomena include a collective group’s influence on the individual members of the group [10]. This personal research strategy culminated in the publication of the edited volume *Fractional Calculus and the Future of Science* [5] in which some of the world’s leading FOC mathematicians were invited to look behind the curtain of mathematics to provide their wizardly advice concerning the future of specific science disciplines of their choice. This was accomplished by peering through the lens of fractional-order calculus and suggesting how what is seen entails a difference in thinking about that specific phenomenon or area of science.

We [11] devised a strategy that provides a way to incorporate complexity into modeling the dynamics of ONs within medicine by utilizing CEs to characterize an array of science principles. Among these are the *principles* of (1) *complexity matching* and *complexity management* (PCMaM); (2) *fractal architecture* (PFA); and (3) *multifractal dimension synchronization* (PMFDS); in addition to several others. We arrived at this strategy by examining the dynamic foundations of CE time series (CETS) and thereby revealing how the statistics of CETS are related to complexity, such as why 1/f-noise is replaced with 1/f-variability and how the diagnosis of illness is being made in an ever-expanding domain of applications. But to appreciate the source of these ubiquitous principles and CEs, we begin with some history of fractality and its implicit as well as explicit relationships with CETS.

### 1.2. Some History of Complexity and FA

In the heady atmosphere of the release of research science from the security constraints of world conflict after the defeat of Italy, Germany, and Japan, the science heroes of World War II began openly discussing and writing about applications of that research to the post-war world and bringing into the light what they had been thinking outside the confines of weapons research. One such discipline was the nascent field of Information Theory (IT) in which two American researchers stand out: the mathematician Norbert Wiener, who synthesized his collaborations with a substantial number of scientists into *Cybernetics* [12], and the engineer Claude Shannon, who did the same with IT [13], with both investigators using the physical concept of entropy to rigorously define a new kind of information. It was this scientific concept of information that guided the molding a new vision of what it is to be human, a vision which in turn is based on the necessity of probability theory for understanding the world of humans and machines, along with their interactions.

This new scientific vision of the transformation taking place in the world around them was clearly expressed by Wiener in *The Human Use of Human Beings* [14]:

...Physics now no longer claims to deal with what will always happen, but rather with what will happen with an overwhelming probability.

      ...It is true that the books are not yet quite closed on this issue and that Einstein (as well as others)...still contend that a rigid deterministic world is more acceptable than a contingent one; but these great scientists are fighting a rear-guard action against the overwhelming force of a younger generation.

      ...In control and communication we are always fighting nature’s tendency to degrade the organized and to destroy the meaningful; the tendency...for entropy to increase.

Cybernetics was a new branch of science whose purpose is to quantify the interface between humans and machines, making explicit Wiener’s belief that the social and life sciences are as lawful as the physical sciences. The past failures of science to find such laws within life science is a consequence of the complexity of the phenomena being studied, not a justification for not seeking them out. Shannon shied away from speculations on the use of IT outside a strict engineering context and indeed often ridiculed those that made them. On the other hand, Wiener’s cybernetics embraced the human potential of the new discipline. It is one of Wiener’s world-changing speculations, its subsequent proof and the understanding it has provided about the increasing complexity in today’s life science, that we address in this tutorial.

The conditions necessary to most efficiently transport information between complex networks and its mechanisms can be traced back to Ross Ashby’s 1957 *Introduction to Cybernetics* [15]. Unlike this earlier work, we argue, both here and elsewhere, that complexity can be expressed in terms of CETS, which are generated by the process of spontaneous self-organized temporal criticality (SOTC) [16], which we explain subsequently after laying a proper foundation. Complex phenomena, appearing in disciplines from anthropology to zoology and all those in between, satisfy the homeodynamic condition and host CEs that we show drive information transport within and information exchange between ONs within the human body as well as between human bodies.

It has been over half a century since Ashby alerted the scientific community that the main difficulty of regulating living networks, which he named *requisite variety,* is the variety of disturbances that must be regulated against. This insightful observation led some scientists to reason that it is only possible to regulate such ONs if the regulators share the same degree of complexity (nonlinear variability) as the ONs being regulated. Herein, we replace Ashby’s term requisite variety with the more encompassing term complexity matching, or complexity management. The *complexity matching effect* (CME), or as it has evolved into the *principle of complexity management* (PCM), has been empirically identified in a wide variety of disciplines since its introduction [17] and interpreted as a new kind of resonance [18] that depends on the entire spectrum of ON interaction and not on a single frequency perturbation. Examples include two-person verbal communication [19], walking rehabilitation of the elderly [20,21], motor control [22,23], and interpersonal coordination [24,25]. The different disciplines that empirically use the PCM to explain their empirical observations lend support for the use of the term ’principle’ in these contexts.

Phenomena requiring the *transport of information* rather than the *transport of energy* for its understanding were believed to be interesting curiosities confined to engineering applications such as communication theory in the limited sense introduced by Shannon and Weaver [26], which appeared at the same time as Wiener’s book on cybernetics. However, the subsequent increased sensitivity of experimental tools, enhanced data processing techniques, and ever-increasing computational capabilities have all contributed to the expansion of STEM research in such a way that those phenomena once thought to be outliers in a statistical sense have transitioned to being the central topics of discussion. These curious processes are now described as exotic scaling phenomena, but as we subsequently discuss, forming a basic understanding of them requires a new statistical perspective, one which is provided by CEs as explained in [11].

### 1.3. Introducing ’Fractal Time’

The American (born Hungarian) mathematician, physicist, computer scientist, engineer, and polymath John von Neumann died in 1957 at the age of 53 and was the developer of the two-step strategy employing a primary and secondary language for the computer that is still used today [27]. The primary is the machine language used for communication and control within the machine, while we humans employ a secondary language to communicate with the machine. Von Neumann suggeted that there may well be a primary and secondary language in the human central nervous system (CNS), emphasizing that the primary language is vastly different from any language then known. His book on the computer and the brain was left unfinished but was eventually published posthumously by his wife. His untimely death prevented him from meeting the person who 20 years later introduced the mathematical concepts that have proven to be foundational for the primary language of the CNS and the mammalian brain: the father of fractals, Benoit B. Mandelbrot.

Just as the physics community had become accustomed to the relativistic space/time view of the universe, Mandelbrot identified yet another twist of time for our consideration with his introduction of fractals and specifically his recognition of the need for a ‘fractal time’ [28]. Fractal time itself, soon after Mandelbrot introduced it into the lexicon of science, became a center piece for wide-ranging research, as in the subjective nature of creativity [29]; the objective nonlinear dynamics [30] to describe the self-similar variations along different time scales producing a frequency (*f*) spectrum having an inverse power law (IPL) power spectral density (PSD) ($f^{-\beta }$); and in life science [31]—wherein it was shown that heart rate variability (HRV) statistics are fractal and interpreted according to the time intervals (τ) between events as being given by an IPL probability density function (PDF) ($\tau^{-\mu }$) accounting for fractal time series, including electroencephalogram (EEG) time series [32]. A fractal time series portends that an IPL PDF is error tolerant, as subsequently discussed for empirical stochastic fractal dimensions [33,34] (see SM2).

Many theoretical constructs of time have been used to explain the passage of chronological time using theory and empirical data together but without critically examining the ‘device’ (instrument) empirically used to interpret that data. The instrument in question is the human brain, and Buzsáki eloquently explains in his chapter *Space and Time in the Brain* [6] that the human brain has no direct experience of time and space but only of duration and motion. He guides us to this conclusion by means of a lucid presentation of how the brain records not only the ‘what’ of an event but the space/time stamp of the ‘where’ and ‘when’ as well as how this information is to be used. He goes on to weave a convincing narrative of why as a neuroscientist he reached the alternative perspective that space and time are “human-invented concepts” that were constructed “outside the brain” by which he meant that physical concepts such as time were constructed independently of any empirical information regarding how the brain operates. In fact, Buzsáki emphasized that physiologists accepted almost without question the objective role of time in their experiments using either the Newtonian or relativistic notions of time. Consequently, all attempts at supporting the above “human-invented concepts” of time using empirical physiologic constructs have been unsuccessfully sought within the brain itself.

A successful conjecture based on empirical data identifies the importance of IPL PSD ($f^{-\beta }$) of EEG time series, which supports the hypothesis of fractal time intervals within the network dynamical explanation of the nearby and global characteristics of brain operations [35,36]. The IPL index β is related to the fractal dimension of a time series, with the latter being a determining factor in how the brain operates, as we shall see.

### 1.4. Questions, Answers, and Hypotheses

The questions we ask as scientists are not nearly as important as what we accept as satisfactory answers to those questions. Herein, the questions of primary interest relate to the nature of time and how its properties determine the operation of the human brain, which remains a mystery because there exists no coherent quantitative predictive theory of how information is generated; organized; or used in cognition, memory, and learning, not to mention how that information is transmitted and stored within the brain. We suggest using what we know about the mathematical properties of fractals to formulate a general hypothesis about the operation of the human brain and test it against the empirical data and predictions entailed by the hypothesis. Consider the FA hypothesis (FAH):

The fractal architecture hypothesis stipulates that ‘fractal time’ determines the complexity of multifractal dimension (MFD) time series and poses the self-similarity in ’structural design’, whereby a ’thing’ is characterized by the magnification of a small part of it being statistically equivalent to the whole.

Therein, the term ‘structural design’ implicitly refers to the three classes of fractals, and ‘thing’ refers to fractal geometric objects, fractal time series, and the fractal manifolds on which chaotic dynamics unfold. The FAH is consistent with building design [37], urban planning [38] and growth [39], metabolic allometry [40], consciousness [41], physiology [42,43], biology and medicine [44], shaping life [45], medical rehabilitation [46], and the all-important explicit concept of ’fractal time’ discussed by Zaslavsky [47]:

*Fractal time* can be considered as one of the most important concepts in the description of fractal properties of chaotic dynamics...A quick way to introduce the notion of fractal time is to consider a set of identical events ordered in time and to apply a notion of fractal dimension to the set of time instants.

This definition of fractal time by Zaslavsky is equivalent to the requirement that a sequence of events be statistically independent of their nearest neighbors and is therefore renewal, as first defined by Feller [48]. The sequence could be Poisson, with the time interval between events being exponential. On the other hand, the independent time intervals between events could be generated by an IPL waiting-time PDF, thereby yielding a sequence of CEs, as described by Cox [49]. A time series X(t) is said to consist solely of CEs, if given a constant λ, the scaling relation $X\left(\lambda t\right)=\lambda^{\delta }X\left(t\right)$ is satisfied, and δ is the scaling index. The scaling index has been shown to be equal to the fractal dimension D (=2−δ), which is consequently a unique measure of the complexity of a CETS.

**Figure 1:**
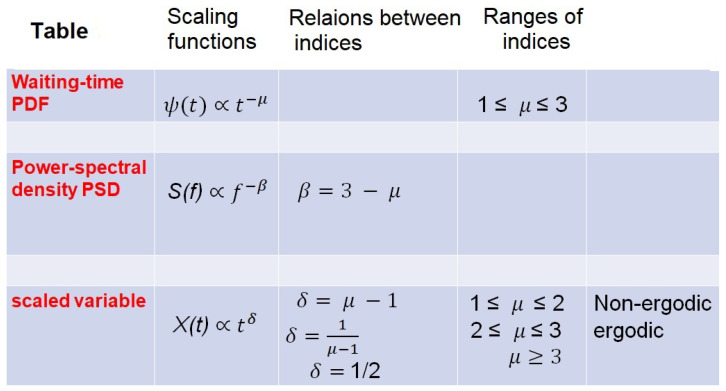
In this table, we record the scaling index δ from the homogeneous scaling relation for the scaled variable X(t), relating it to the IPL power spectrum index β through the waiting-time PDF ψ(τ) IPL index μ. The value μ=2 is the boundary between the underlying process having a finite (μ >2) or an infinite (μ <2) average waiting time and is also the point at which β=1, where the process is that of true 1/f− noise. Consequently, β and μ are interchangeable measures of complexity. For an ergodic time series such as that determined by the waiting-time inverse power-law, index μ increases with decreasing scaling index δ, and the fractal dimension increases. Adapted from [1] with permission.

Figure 1 is copied from West et al. [46] and provides easy reference to all the derived relations among the IPL scaling indices for the PSD index β, the IPL waiting-time PDF index, μ and the scaling index δ for the scaled variable X(t), as well as the scaled PDF. For example, suppose the trajectory $X\left(t\right)$ crosses a known level at a specific time and we want to know how long we must wait to recross that same level. Given that the waiting-time PDF has the IPL form, we denote the generic IPL index for the waiting-time PDF μ by the symbol $\mu_{D}.$ From the parameter relations in Figure 1, it is clear that it is possible to prove that the IPL index is equal to the fractal dimension so that we obtain the following [46,50]:

(1) $$\mu_{D}=D=2-\delta .$$

On the other hand, it is possible to establish using the probability of crossing and recrossing any fixed value of the diffusion trajectory which has been shown to also be IPL but with an Index $\mu_{S}\neq \mu_{D}$ (see SM1).

The FAH is foundational because observation verifies that it properly entails the generation, structuring, and transport of information within an ON such as in the synchronization of the time series datasets of the mammalian brain’s EEG (B), the ECG of the human heart (H), and the respiration of the lungs (L) time series [1,2,51], which are referred to as the HBL-triad as reviewed in Section 5.4 and with further details presented in SM3. The neuroscientist Buzsáki did in fact implicitly introduce a version of the FAH to explain the empirical observations of a functioning mammalian brain. He also makes several prescient conjectures in *Rhythms of the Brain* [35] regarding what is entailed by FAH for the structure of the mammalian brain and its neuronal components. Much of the empirical evidence supporting the FAH in a broader medical context appeared after the publication of Buzsáki’s book [35]; much of this support is scattered throughout the literature [52] and has been pulled together and discussed in *The Fractal Language of Medicine* [53].

### 1.5. Background on Crucial Event Time Series (CETS)

Many complex natural and man-made processes are aggregated into collective events that signify typical behavior. Examples from the physical domain are waiting in queues and earthquakes [54]; the life sciences give us heart rate variability (HRV), stride rate variability (SRV), and breath rate variability (BRV) [28,55]; the social domain offers uneventful demonstrations, terrorism, and wars [56]. No matter how complex the underlying dynamics involving the interactions among fundamental elements of a network being studied are, the resulting emergent behavior can be viewed as the unfolding of particular events localized in time. In this way, the stochastic behavior of a time series is characterized by a sequence of events described in terms of a waiting-time (sojourn) PDF ψ(τ) where, for example, τ is the time interval between beats of the heart, the interbreath intervals of the lungs, or the turn-taking times between two individuals deep in conversation. Each of the event processes mentioned here, as well as dozens if not hundreds of others, fall into the category of crucial event (CE) [11] time series (CETS).

CETS are members of a larger class of events called *renewal* in which a sequence of events occurs at times $t_{1},t_{2},\cdots$ and are such that the time interval between successive renewal events (REs) is denoted by τ so that the REs occur at times $t_{1}=\tau_{1},\;$$t_{2}=\tau_{1}+\tau_{2},\;$$t_{3}=\cdots$ and are renewals consisting of those events which reset the clock to that of the initial state of the generating system after their occurrence [57]. The set of times {τ} for the REs are consequently statistically independent. REs found in physical systems include blinking quantum dots [58] and defects arising in the weak-turbulence regime of liquid crystal [59]; in medical systems, these include the anomalous diffusion of tagged particles inside living cells as well as in all physiological time series, and in social networks, these include the influence of zealots (leaders?) on group activity [10]. The list can be extended to virtually every network, including networks-of-networks, and in every discipline that needs to exchange information among networks in order to fulfill a function.

It follows from this observation that network A must share identical coding and decoding capabilities with network B in order for network B to most efficiently decode the message being delivered to it by network A. In plain English, the two networks must speak the same language in order to communicate efficiently with one another. When this banal observation is applied to the operation of the human body, it leads to the remarkable implication that the ONs within the human body must share a common language. The brain, heart, lungs, spleen, gut, and so on must therefore all share this same language in order to carry out their coordinated tasks. But surely that cannot be right given the variability in the time intervals witnin a single CETS, can it?

**Figure 2:**
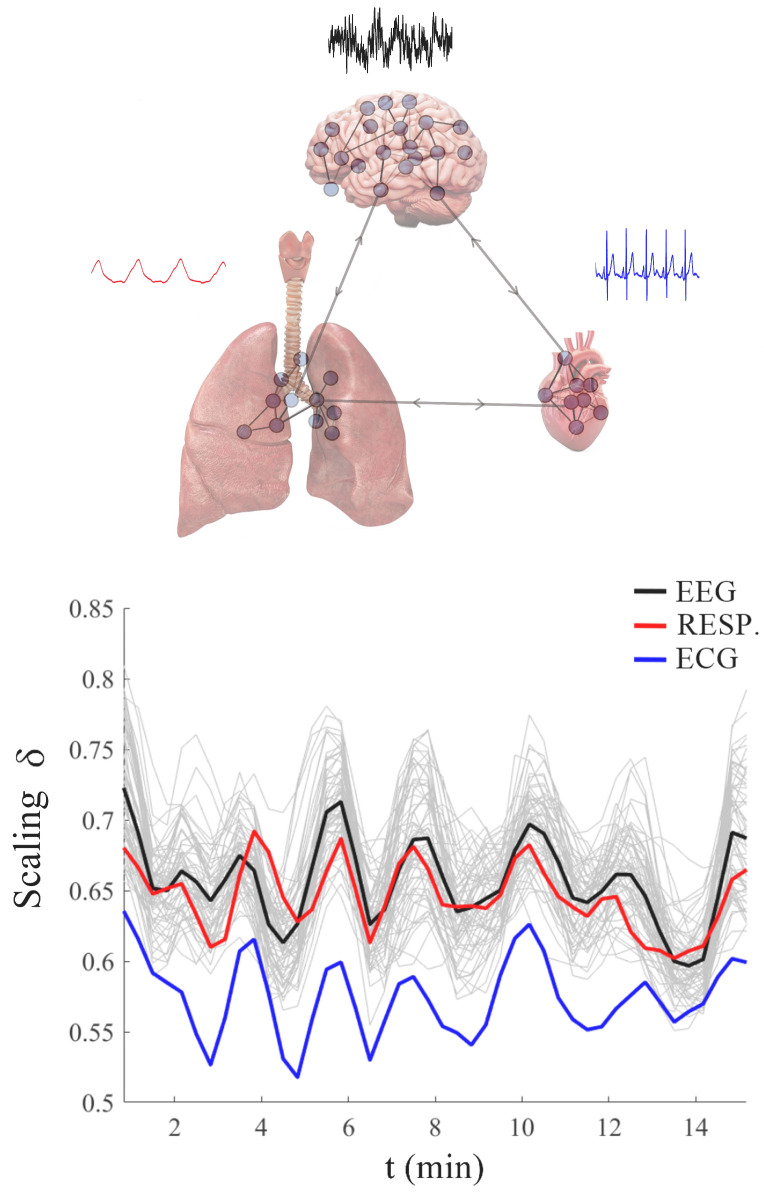
The upper panel depicts the mutual interactions among the brain, heart, and lungs, along with approximatley five seconds of simultaneously recorded time series. The ONs are indicated by cartoons, but the time series are the actual empirical time series. One would be hard pressed to convincingly argue that these three simultaneously recorded time series are in resonance with one another. But that is one of the goals of this tutorial. The lower panel is the measure of the 66 scalling indices $\delta_{j}\left(t\right),\;$j=1,2,⋯,66 and are all seen to be quasiperiodic. A complete discussion of the interpretation of these results are given in the text; see Section 5.4 and SM3.

Just look at the difference in the number of scale sizes in the time series shown in Figure 2 for the HBL-triad. Can the three time series in the HBL-triad as well as all the other time series generated by the other physiologic ONs be in a new kind of synchronization? The surprising answer is yes they can, as we explained elsewhere for the first time using empirical data [1,2,51], and in this tutorial, we pull together all the contributing strands and reveal the fractal tapestry underlying Nature’s greatest accomplishment: that being the cognitive capability of human beings.

It is important to understand the dynamic origin of CEs, as well as the significant role they play in the exchange of information among ONs [2]. CEs are a manifestation of cooperative interactions among the basic units of an ON that spontaneously organizes itself and has been referred to as self-organized criticality (SOC) in the network science literature. We have come a long way in our understanding of SOC since the original work of Bak et al. [60] over a quarter century ago, including a new approach to SOC that emphasizes the temporal and not the intensity PDF [16,61]. Mahmoodi et al. [16] identified this manifestation of spontaneous self-organization in time as self-organized temporal criticality (SOTC) in terms of the CEs just defined, namely, the events that the authors of [62] were able to find in heartbeats and which occur on an intermediate time scale after an initial transient regime to the condition of intermediate asymptotics. The three time regimes of intermediate asymptotics identified by Barenblatt [63] and implemented in the SOTC are a form of variability that we subsequently connect to the physiological variability that led Allegrini et al. [62] to their diagnostic insights which we subsequently review herein.

STEM investigators have often taken waiting-time PDFs to be exponential, which is not surprising, since this frequently made assumption defines a renewal process that is Poisson in time and which is general and straightforward to implement. On the other hand, observing empirical ONs, one invariably finds that the statistics are heavy-tailed distributions (HTDs) which define a distribution class to which the Pareto, Lévy, Log-normal, Mittag–Leffler, Hyperbolic, and all the other IPL PDFs belong. We assert that the statistics most consistent with observed PTS have IPL PDFs in time which are also renewal [49,57] and constitute CETS. The latter theoretical result is often obscured by noise, and other HTDs have been used to explain the observed data; see, e.g., the use of tempered Lévy statistics to mistakenly describe the large-scale fluctuations in HRV time series [64,65]. A technique involving fractional-order calculus (FOC) avoids a number of the technical problems encountered in these and subsequent studies cited in [53,66] resulting in a physiologically consistent model of cardiac control of healthy HRV [46].

Consequently, an empirical time series consisting of a mixture of the two types of renewal processes requires that we think differently about how to process the dataset in order to determine whether the recorded events are Poisson events or CEs. This becomes even more challenging when the Poisson process is modulated and produces an IPL PDF that appears indistinguishable from the PDF for CEs but, as it turns out, can be distinguished from it using modified diffusion entropy analysis (MDEA) (see the step-by-step approach to using MDEA in SM3). Scafetta and Grigolini [67] developed DEA in dealing with empirical time series that has turned out to be an important data processing tool, as we subsequently show.

## 2. Math Modeling of Medical Phenomena: A Primer

The goal of the present section is to facilitate fertile crosstalk between the academic communities that comprise physicians and medical professionals who are neophytes in the mathematical modeling of physiological time series, as well as mathematicians and physicists who are embarking on their maiden voyage of applying the FAH to the analysis of physiologic datasets to generate practical solutions that address unmet patient needs in medicine. The comments in this section are for those not having mathematics as a second language in science.

**Criticality can ferment practical applications of FAH:** The optimal fitness of an ON requires maximizing the ability to be robust against perturbations and to be strongly adaptive in the face of environmental uncertainty to the extent that the biophysical constraints of an organism allow.

Our working definition of health is a state of being optimally adaptive alternatively recognized as the state of optimal complexity in direct corrrepondence of Ashby’s requisite variety, while sickness can be defined as a state where this adaptive capacity has been eroded as measured by the lowering of complexity below the optimum [68,69]. Moreover, a healthy body is always adapting, which is to say that it is in a state of never-ending fluctuation or of maximum complexity [17,70]. The FAH—particularly regarding the notion of criticality–provides us with an analytical framework that holds the promise of transforming these definitions of health vs sickness into practical tools that can improve bedside care by addressing unmet clinical needs.

**Criticality provides courses of action for ON response:** Criticality in the sub-dynamics of the human body arises in the context of switching communication strategies among the nodes (ONs) that comprise the body’s NoONs. The capability to switch communication strategies—that is, the dynamics of information flow within the network (NoON—“ *the software*”)—while preserving the node structure (ONs—“ *the hardware*”) ultimately confers an ability to adapt to uncertainty. In physics jargon, each communication strategy can be called a phase, and thus, switching among various communication strategies can be called *multiple phase transitions*.

Optimal health can thus arise when the body has the following [46]:

(1) A maximal number of phases;
(2) The ability to rapidly switch between phases;
(3) The ability to match the chosen phase to a required adaptive response.

The ability to carry out step (2) requires being close to the necessary phase transitions as this can enhance adaptive versatility. In the physics literature, this condition requires being sufficiently close to criticality. Analyzing a physiological signal processing paradigm grants us a way to identify when a biological network is close to criticality (and when it is not). Injury or illness can potentially result when there is a negative disruption of (1), (2), or (3).

**Fractal time enables multiscale network synchronization**: A key aspect of the body is that each of its nodes is a nested network. For example, intra-cellular networks of nucleic acid communication give rise to intra-cellular protein networks, which ultimately can give rise to inter-cellular communication networks, ultimately giving rise to ONs and to NoONs. Consequentially, each of these ONs in the hierarchy communicates on a set of different time scales as determined by their scaling index δ. For instance, intra-cellular networks will likely transmit signals over a shorter time interval compared to inter-cellular networks due to the differenes in distances involved. Thus, a given measurable physiological response is the result of a large number of networks coordinating their actions across multiple different time scales.

This massive coordination requires that our notion of time be reframed to account for these multiple-scale coordinations. The multifractal dimension (MFD) of each ON time series is a measure of the time-dependent complexity of that ON time series, and it is the matching of the MFD ON time series that provides the synchronization referred to as CS [66]. We call this more precise definition *fractal time*.

### 2.1. FA Entails Different Thinking Modes

We focus here on the different ways CEs enable us and indeed force us to think about complex phenomena in the physical, life, and social sciences. The complexity of nonlinear dynamic processes demand that we extend our functional horizons beyond the analytic and into analyses suggesting that the functions of interest lack traditional equations of motion [5]. To explore this lack of traditional support, we introduce fractal architecture (FA) into the ways we considier how Nature has overcome adversity to regain healthy function, particularly through the use of fractal structures, fractal statistics, and fractal dynamics. *The method we have used is to never impose fractality without an obvious empirical invitation but when it is found in the data to determine which of the many fractal functionalities explains its presence and only then to develop a workable and verifiable fractal model of the structure, the dynamics, or the statistical behavior of the phenomenon under study*.

Herein, we focus on the different ways CEs enable us to think about complex phenomena, particularly in the medical implications within life science. In [4], we explored the more obvious mathematical reasons why traditional statistical processes, including those described by integer-order probability calculus (IOPC), are not sufficient to capture the full range of dynamic behavior found in natural and human-made processes and events. Specifically, the complexity of nonlinear dynamic processes demand that we extend our horizons beyond analytic functions and analyses suggesting that the functions of interest lack traditional equations of motion [5]. To explore this lack of traditional dynamics, we introduce fractal thinking as a kind of in-between thinking; between the integer-order moments, such as the mean and variance, where are fractal moments required when empirical integer moments fail to converge; between the integer dimensions, there are the fractal dimensions that are important when datasets have no characteristic scales, and between the integer-valued operators that are local in space and time, there are noninteger operators necessary to describe dynamics that have either long-time memory, spatial heterogeneity, or a combination of the two [2]. Understanding complex phenomena requires new ways of thinking and, in addition to the FOC the mathematical properties of CETS, we must add another ‘dimension’ to the framework for that thinking.

**Figure 3:**
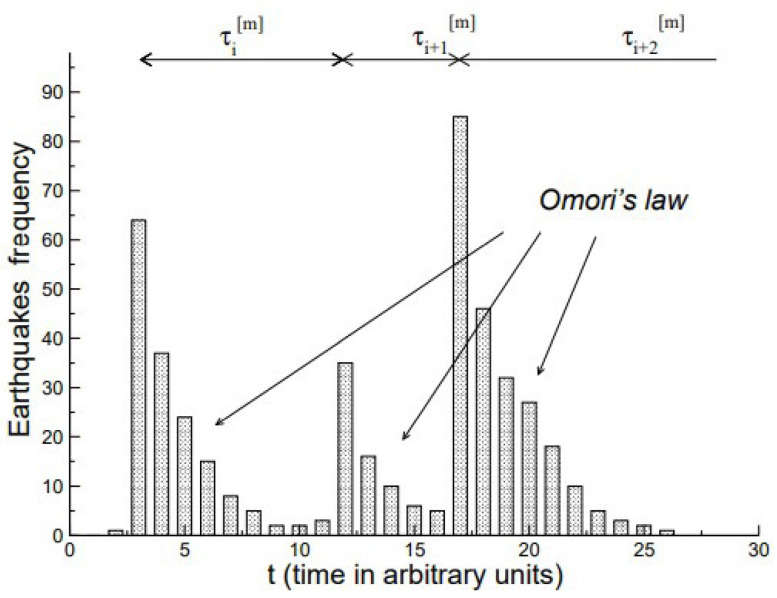
We observe a frequency peak for each main shock followed by an after-shock swarm, whose peaks decay according to Omori’s law; see text. The horizontal dotted arrows indicate the time intervals $\tau_{i}^{\left[m\right]}$ between consecutive main shocks. The MDEA described in the text and in SM3 provides a technique for obtaining information on the PDF of these time intervals. From [54] with permission.

The analysis of the distribution of seismic fluctuations depicted in Figure 3 serves as an exemplar for the more general purpose of detecting the statistical properties of CETS that are not visible. To be clear, CEs are renewal events that directly cause other events to come into existence and whose origin would be predictable if the time occurrence of their causes were known, which they are not. Renewal is mentioned as a statistical term meaning that the time intervals between succesive CE are independent and which are more fully described as the need arises. By invisible CEs, we mean CEs embedded in a sea of non-CEs, the latter being either initiated by environmental fluctuations or caused by the subsequently invisible CEs themselves. These secondary events camouflage the CEs, making it difficult to detect them with any degree of accuracy. We have briefly discussed the properties of CEs, regardless of whether they are visible or invisible, and subsequently address the extent to which the CEs are predictable rather than being completely random.

To facilitate subsequent theoretical discussion of various kinds of memory, a few general remarks are in order. These remarks are made in the context of an example concerning the frequency distribution of the magnitudes of earthquakes depicted in Figure 3. Recent geophysical observations indicate that main fracture episodes can trigger long-range as well as short-range seismic effects. Mega et al. [54] point out that earthquakes are grouped into temporal clusters of events, and these clusters are *unorrelated* from one another, but the *intra-cluster shocks are correlated* in time as given by Omori’s law. This empirical inverse power law (IPL) states that the main shock, i.e., the highest magnitude earthquake of the cluster occurring at time $t_{0}$, is followed by a swarm of correlated earthquakes (aftershocks) whose number (or frequency) n(τ) of decays in time $\;\tau =t-t_{0}$ from the main shock is defined as an IPL. The waiting time between sequential seismic events is determined by the waiting time IPL probability density function (PDF), with the IPL index close to unity in this case.

Like Omori’s law for the swam of aftershocks, empirical PDFs are often defined through the IPL with diverging first and second moments. The IPL PDF is the backbone of complex dynamic networks and is particularly important in characterizing the time interval between CEs in an empirical CETS. The IPL index μ is a parameter measuring the level of complexity of the network, and in this context by complexity we mean the ability of a network to self-organize into metastable structures. Moreover, these structures can survive for long time intervals, thereby introducing long-time correlations. Paradisi et al. [71] discuss the spatial correlations induced by such metastable structures in the context of vortex motion in turbulence. They also point out that the limit of large μ values is associated with a weak coupling in the nonlinear interactions and consequently with a low level of self-organization. On the other hand, decreasing μ increases the coupling strength along with the level of self-organization. This is consistent with our interpretation of μ serving as a measure of complexity and thereby being labeled as the complexity index.

### 2.2. FA and Anomalous Diffusion

**Figure 4:**
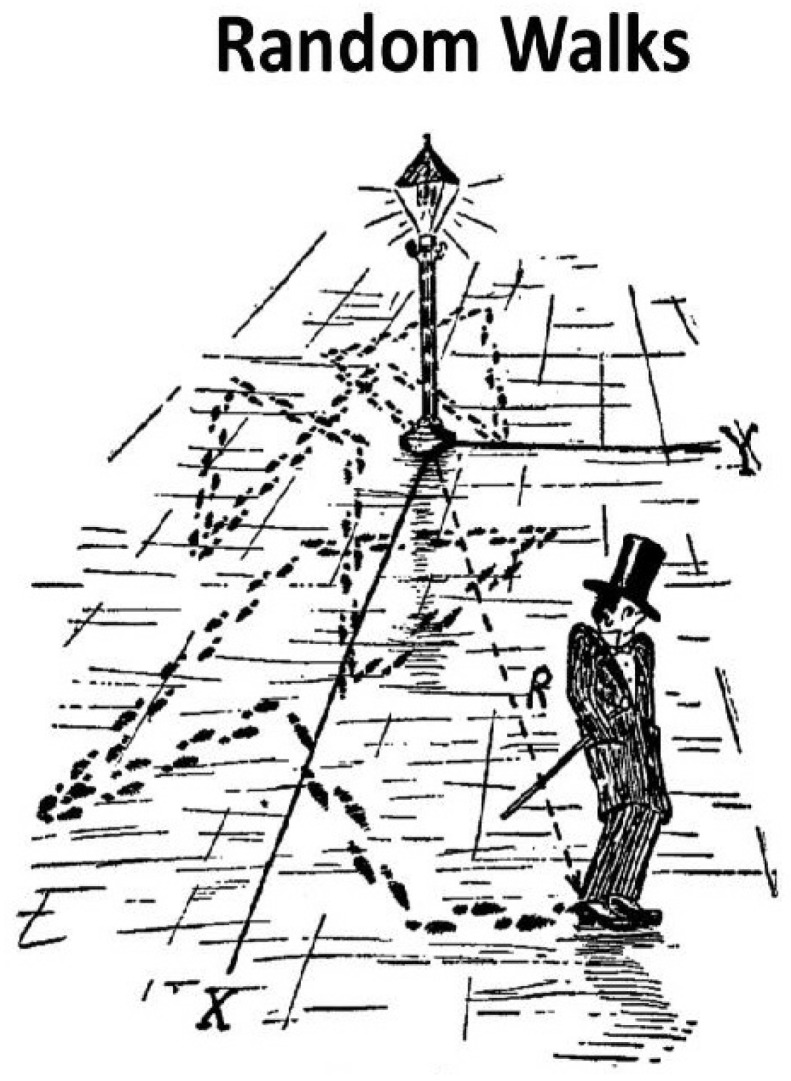
Cartoon of a 2D random walk (RW), otherwise known by the more colorful name of drunkard’s walk. Taken with graditude from Gamow [74] as well as the quotation taken from the Preface: “The time has come”, the Walrus said, “To talk of many things,...” [75]...of atoms, stars, and nebulae, of entropy and genes, as well as whether one can bend space and why the rocket shrinks.

The definition of a CETS is straightforward but what it entails is not, and for that reason, we review some of the physics-based statistical concepts necessary for its understanding. Most students of the physical sciences encounter statistics for the first time in the study of simple diffusion, which is used to derive the Gaussian PDF. The argument producing this PDF was first given in the form of a random walk (RW) by Lord Rayleigh [72] in 1905 the same year Einstein [73] explained molecular diffusion using the precursor to the probability calculus formalism. The random walker’s displacement is updated as depicted in Figure 4 at step *n* given by $Y_{n}$ by adding a random number $\eta_{n}$ to it and obtaining $Y_{n+1}=Y_{n}+\eta_{n}$. The RW process can be formally expressed as a discrete dynamic process, using the downshift operator *B*, as $\left(1-B\right)Y_{n+1}=\eta_{n}$. After *N* steps, the total displacement is $Y_{N}=\eta_{1}+\eta_{2}+\cdot \cdot \cdot +\eta_{N}$, and for *N*, it is →∞ when the random variable η(t) is a continuous Wiener process, where the displacement PDF is known to be given by the continuum form of the Gaussian: $P\left(y,t\right)=\exp\left[-\frac{y^{2}}{2\sigma^{2}\left(t\right)}\right]/\sqrt{4\pi \sigma^{2}\left(t\right)}$, with a variance that increases linearly with the time $\sigma^{2}\left(t\right)\propto t$, that is, the displacement of the random walker increases linearly with the square root of time $Y\left(t\right)\propto \sqrt{t}$.

Hosking [76] generalized this simple RW to ${\left(1-B\right)}^{\alpha }Y_{n+1}=\eta_{n}$, the fractional RW (FRW), where the index α is not an integer, and established that the operator ${\left(1-B\right)}^{\alpha }$ has an inverse given in terms of a binomial expansion. Consequently, the total displacement after *N* steps is determined by the accumulation of *N* independent random events stretching infinitely far back in time with their relative impact determined by the ratio of gamma functions. As the step index in the binomial expansion (discrete time) *k* becomes large, the ratio of gamma functions in the binomial expansion becomes proportional to $k^{\alpha -1}$, which is an IPL, since 0<α≤1/2, as determined in the analysis. Note that since the total displacement is linearly related to the random events, the PDF remains Gaussian; however, $\sigma^{2}\left(t\right)$ is no longer linear in time, but it varies as the IPL $\;t^{2\alpha -1}$. Although these arguments are based on a FRW, this is our first indication that fractional dynamics are connected to temporal complexity; see West’s lecture notes [77] and SM1 for details.

The scaling of the solution indicates that the FRW generates a random process with memory, and consequently, a new RW can be generated using the random events with memory $\left(1-B\right)X_{n+1}=Y_{n}$. The solution to this latter RW in the continuum limit has the stationary auto-correlation function $C\left(\tau \right)=\langle X(t+\tau )X(t)\rangle$$\tau^{2H}$. Mandelbrot [28] introduced the scaling exponent *H* to honor the civil engineer Hurst who first used this scaling index in the study of overflow of the Nile River statistical time series.

**Fractional Brownian Motion (FBM):** One of Mandelbrot’s first applications of the fractal concept to statistics was in the context of Brownian motion. He in collaboration with van Ness to create the concept of fractional Brownian motion (FBM) as an extension of the random displacement X(t) of a Brownian particle by generalizing the Hurst scaling exponent *H* from the single value of 0.5 to the range of values 0<H<1 [78]. The second moment of an FBM process initiated at time $t_{0}$ diverges as ${\left|t-t_{0}\right|}^{2H}$ such that H=0.5 is the singular case of independent displacements valid for Brownian motion, and processes for H≠0.5 are properly fractal.

FBM is not compatible with equilibrium statistical physics but is based on the stochastic rate equation for free diffusion:

(2) $$\frac{dX(t)}{dt}=w\left(t\right)$$

The stationary auto-correlation function for the noise $\eta \left(t\right)$ has a short-time correlation for ordinary diffusion, which is replaced in FBM by $w\left(t\right)$, which has a stationary but nonintegrable auto-correlation function with a diverging correlation time. The FBM proposed by Mandelbrot, with a vanishing initial state, yields the correlation coefficient:

(3) $$r=\frac{\langle X(-t)X(t)\rangle }{\langle X{\left(t\right)}^{2}\rangle }=1-2^{2H-1},$$

and as Feder [50] emphasizes, for H=0.5, the correlation of *past and future* increments r=0 for all *t* yields an uncorrelated random process. However, for H>1/2, the process is persistent, indicating that an increasing (decreasing) trend in the past entails an increasing (decreasing) trend in the future for all *t* as indicated by the positive correlaton coefficient r>0. On the other hand, for H<1/2, the process is anti-persistent, indicating that an increasing (decreasing) trend in the past entails a decreasing (increasing) trend in the future as indicated by the negative correlation coefficient r<0.

Note that the auto-correlation function given by Equation (3) for FBM is in direct conflict with what is normally assumed or can be proven from the statistical records of physical networks. Thermal equilibrium requires that events correlated when separated in time by Δt become uncorrelated in the limit Δt→∞, which is certainly not true the case above. Moreover, in a second-order phase transition, e.g., as the critical point of a fluid is approached from above the critical temperature, the fluid density auto-correlation function transitions from being an exponential with independent increments to being an IPL with a long-time correlation.

Mannella et al. [79] emphasized that there exist anomalous forms of diffusion signaled by H≠0.5, departing from the Gaussian assumption of Mandelbrot. This is a consequence of a confusion between two forms of deviation from ordinary diffusion. The deviation due to CEs may also deviate from the Gaussian assumption. Culbreth et al. [80] established a clear distinction between two processes yielding anomalous diffusion and 1/f-fluctuations. The first is stationary FBM using stationary correlation functions, and the second rests on the action of CEs generating the breakdown of ergodicity and an effect named *aging* FBM (AFBM). They [80] showed that although the joint action of CEs and non-CEs may have the effect of making the CEs invisible, an entropy approach to data processing enables, in spite of their not being visible, the detection of their action, as we subsequently show.

### 2.3. Multifractal Dimensions (MFDs)

It is worthwhile to recall that the name FBM was coined in the classic paper by Mandelbrot and Van Ness [78], and the name *fractional* was adopted because they made use of the FOC in their definition of a FBM time series $B_{H}\left(t\right)$:

(4) $$B_{H}\left(t\right)=\underset{-\infty }{\int^{\infty }}\left[{\left(t-\tau \right)}_{+}^{H-1/2}-{\left(-\tau \right)}_{+}^{H-1/2}\right]dB\left(\tau \right),$$

where dB is a differential Wiener process. However, the process was first introduced in 1940 by Kolmogorov [81], but as pointed out by Taqqu [82] in his tribute to Mandelbrot, it is undoubtedly the seminal paper of Mandelbrot and Van Ness which put the focus on fractional Brownian motion and gave it its name. Although they introduced the FOC into the discussion, the two of them did not believe that it was sufficiently significant to develop the interpretation further given the context of its utilization and their interpretation of the integral in Equation (4) in terms of a moving average. The fractional operator they used in the definition of (stationary) FBM had been defined earlier by Weyl in 1917:

(5) $$X\left(t_{1}\right)-X\left(t_{2}\right)=\sum_{j=1}^{3}\int_{-\infty }^{t_{j}}\frac{{\left(-1\right)}^{j+1}dB\left(t\right)}{{\left(t_{j}-t\right)}^{1-\alpha }},$$

where again, *dB* is a Wiener noise process, and α=H+1/2. They pointed out that the properties of FBM defined by Equation (5) differ in significant ways from ordinary diffusion depending on the value of the scaling parameter, as we have indicated.

One property of FBM is self-similarity where, like a fractal, for a constant λ, a dynamic variable satisfies the scaling relation:

(6) $$X\left(\lambda t\right):=\lambda^{H}X\left(t\right),$$

which is true of the PDF and not of the individual FBM trajectories and has statistical self-affinity to a mathematician but is referred to as statistical self-similarity by most physicists. Thus, FBM has three properties: (1) has a Gaussian PDF with zero mean; (2) is stationary; and (3) is self-similar with an index 0 < H< 1. Because of these properties, the FBM displacement increases as $X\left(t\right)\propto t^{H}$; which includes the case of simple Brownian motion for H=1/2.

It has also proven valuable to further extend the fractal measure of complexity by changing the fractal dimension of a time series over time, thereby associating a time-dependent fractional dimension or an MFD with the time series. Experiments that stimulate fractal tapping by means of a metronome have provided significant insight into the control of body movements, e.g., see Deligniéres et al. [83,84]. The most familiar body movement is regular walking, which turns out to be not very regular. The variability in stride, the time interval between successive heel strikes, was first recognized in the 19th century [85] but argued to be inconsequential, and therefore, its irregularity was not quantified for nearly 120 years [86]. Deligniéres and Torre [87] determined that the power spectral density (PSD) for the time fluctuations in stride intervals is an IPL.

**Figure 5:**
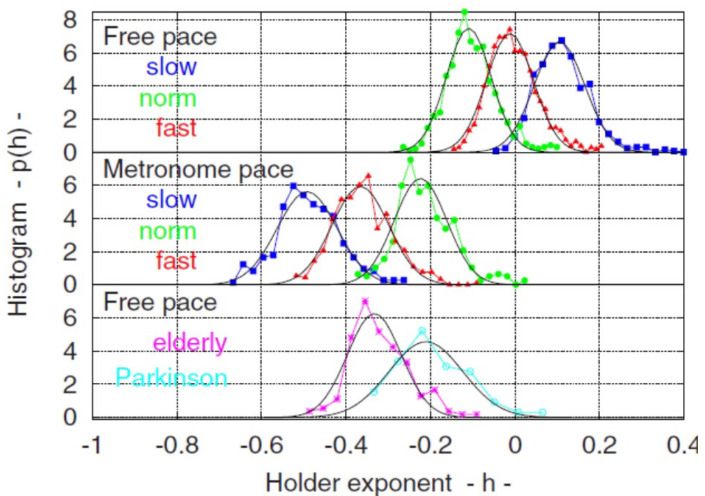
The typical Hölder exponent h=H−1 histograms for the stride interval series under free walking as well as the metronome driving conditions for normal, slow, and fast paces, for elderly and for a subject with PD. The histograms are fitted with Gaussian functions. From [88,89] with permission.

Scafetta et al. [88] point out that walking is accomplished by the two-way exchange between the muscles receiving commands from the nervous system and sending back sensory information that modifies the activity of the central neurons. The coupling of these two complex networks produces a fluctuating stride interval time series that is characterized by MFD properties. These properties depend on several physiological and stress constraints, such as walking faster or slower than normal, as depicted in Figure 5, as well as age and pathology.

As summarized in [11], the multifractal nature of the stride interval fluctuations become slightly more pronounced under faster or slower paced frequencies relative to the normal paced frequency of a subject, as depicted in Figure 5. The subjects were asked to synchronize their gait with the frequency of a metronome, and the randomness increased. An increase also occurred when the subjects were elderly or suffering from neurodegenerative disease, such as Parkinson’s disease (PD). The supercentral pattern generator (SCPG) model of West and Scafetta [89] was able to reproduce these known properties of walking, as well as to provide physiological and psychological interpretations of the model parameters. The control of SRV as indicated by the changing width of its empirical MFD is distinctly different from that observed, for example, by the PSD associated with HRV, as discussed using a Langevin equation to determine the MFD width of the PSD [46].

Ivanov et al. [90] were the first to establish that heart rate variability (HRV) time series have a multifractal spectrum and that the width of the spectrum could serve as a diagnostic of health. They analyzed the heart beat data of several patients using wavelets and determined that healthy subjects have a significantly broader multifractal spectrum than those with a cardiac pathology. This encouraged Bohara et al. [91] to study the connection between multifractality and the CEs in HRV time series. Their study proved that increasing the percentage of Poisson events hosted by heart beats has the effect of making their multifractal spectrum narrower, thereby establishing a dynamic interpretation of multifractal processes that had been previously overlooked.

**Figure 6:**
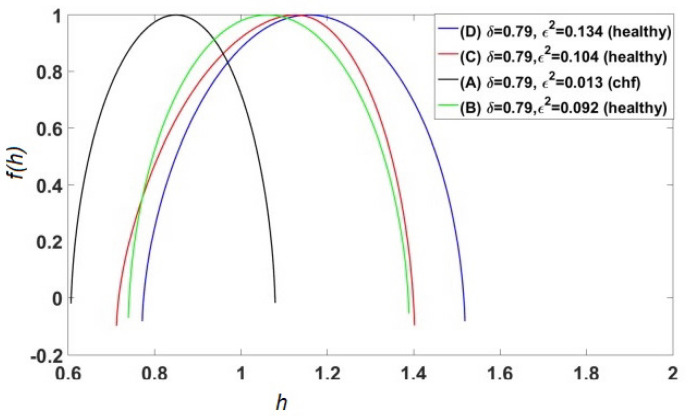
Multifractal spectra of HRV as a function of (see Figure 12 [42]) at a constant value of the scaling parameter δ=0.79: Here, the narrowing of the spectrum for individual A with congestive heart failure is evident. From [91] with permission.

Bohara et al. [91] focused on the individuals labeled A, B, C, and D in their Figure reproduced herein as Figure 6. These patients had the same value for the scaling index δ=0.79, and the distinction between healthy and sick individuals is due to the fact that the heartbeat of the sick patients was affected by excessive randomness [62], as measured by the probability that the detected event is a CE given by ϵ. The empirical source of this parameter will become clear once we have discussed the theory underlying the data processing technique used in the analysis. Whereas, according to Ivanov et al. [90], the distinction between time series from healthy and diseased individuals is indicated by the fact that healthy patients have broader multifractal spectra. Figure 6 indicates that moving from the sick to the healthy patients effectively increased the width of the multifractal spectrum, thereby fully confirming the hypothesized connection between characterizing the HRV dataset as a multifractal spectrum in three dimensions and a point in the two-dimensional (δ,$\epsilon^{2}$) plane. The connection between these these parameters in terms of CEs is made clear subsequently (see also SM1).

A key takeaway message from the remarks so far made is the realization that a great deal of what has been mistakenly identified in the past as noise in time series is actually how control is encoded and communicated between and among complex networks. The time series of interest consists of a sequence of CEs, which is shown to carry information from one complex network to another and to subsequently control its operation. An example from physiology/sociology may help to clarity what is meant.

Two people walking together unconsciously synchronize their gait, even though the gait of each individual is not regular, but has fluctuations in the length and timing of each stride. The apparently random fluctuations in the step-to-step timing during normal walking actually carries information about the correct operation of the motor control network. This information, in addition to locking together the stride patterns of individuals walking together, has been shown to be communicated during arm-in-arm walking from the gait of a relatively young rehabilitation therapist to the gait of an elderly patient consequently improving the gait of the latter [20,21], i.e., information is transferred from the information-rich healthy gait network of the therapist to the information-depleted pathological gait network of the elderly person, resulting in the elderly person regaining a healthy gait pattern.

We show in due course that sequences of CEs appear to be the generic mechanism for how complex networks in physiology and sociology have evolved to control network variability and consequently network stability in order to satisfactorily carry out their function. This is less a teleological statement than a recognition of the fact that the individual mechanisms giving rise to the observed statistical properties in various physiological networks are very different, as are those in social networks. On the other hand, the time series for sub-networks in both physiology and sociology scale in the same mathematical way so that at a certain level of abstraction, the separate mechanisms cease to be important, and only the relations matter independently of the things being related [92]. All this and more is entailed by the principle of complexity management (PCM), as we subsequently show.

CEs are members of a large class of events having the property of being renewal. A sequence of renewal events consists of those events that reset their clocks whenever the generating network randomly initiates a new initial state of the sequence independently of prior initial states. Renewal events in medical networks give rise to the anomalous diffusion of tagged particles inside living cells [93], and in social networks, they describe the influence of group activity on individuals [10]. An example of the importance of being able to identify such a sequence is given by the ability to discriminate between a healthy and a pathological HRV time series, as we continue to show.

Investigators have often taken waiting time PDFs to be exponential, which defines a Poisson process known to be renewal. On the other hand, complex networks often, if not always, have IPL PDFs in time which are also renewal. Consequently, empirical time series are found to consist of a mixture of the two types of renewal processes, and their understanding requires that we think differently about how to process such datasets in order to determine whether the recorded events are predominately Poisson or CEs. This becomes even more challenging when the Poisson process is modulated and produces an IPL PDF that appears indistinguishable from that for CEs alone. The mixed case is the more important situation in dealing with empirical time series, since it can make the CE contribution to the time series invisible.

Thus, a pathology such as congestive heart failure is manifest in cardiac time series in several ways, two of which form the axes in the diffusion entropy analysis (DEA). The scaling of CE time series occurs on an intermediate time scale after an initial transient regime to the condition of intermediate asymptotes using DEA and yielding the scaling index δ. The memory in the time series is measured by the probability that a measured event is non-CE as determined by ϵ. This same technique is subsequently shown to diagnose the extent of other pathologies, including, but not limited to, cardiac autonomic neuropathy (CAN) [94], interpersonal coordination [24], interpersonal finger tapping [22], and the war against terrorism [56].

**Statement of Unmet Need:** Physiological time series (PTS) are a mixture of dynamics that correspond to CEs and non-CEs. Elucidating the dynamics from raw PTS patient data requires a signal processing strategy that can discern between CEs and non-CEs [95]. A signal processing strategy that does not explicitly make this discernment can obscure real-time understanding of pertinent inter-organ dynamics in both health and disease, thereby limiting the clinical utility of PTS. Explicitly using a fractal signal processing strategy can directly address a capability gap in PTS interpretation, which can potentially be a foundation for improving clinical decision making.

**Given Context:** Progressively mounting evidence indicates that the dynamics of physical system interactions (i.e., language) take on a fractal mathematical structure. The *von Neuman hypothesis* that there is an invariant “primary language” of the CNS was later expanded upon by Mandelbrot’s work on ubiquity of fractals in [28]. This primary language of physical system interaction is hence fractal. Physiological systems are no different, involving physical system interactions across temporal and spatially nested processes. Ultimately, this motivates the need to conceptualize organ networks (ONs) and networks among these ONs.

In support of this interpretation, we point to the recent work on Complexity Synchronization wherein a similar signal morphology in the scaling parameter—despite coming from morphologically distinct raw PTS data—corroborates the role of fractal signal architecture as the underlying “primary language” [2,51]. PTS, e.g., the HBL-triad of signals, offer a window into real-time dynamics of ONs and NoONs.

**“People ’hire’ products to get a job done”:** [96] The goal of the “job” is to reduce uncertainty with interpreting real-time dynamics captured by raw PTS data. A fractal signal processing strategy has the potential to address the above goal compared to incumbent paradigms. This offers a sustained competitive advantage in the “conceptual market place” of signal processing paradigms. Incumbent paradigms analyze data based off of assumptions (e.g., a CE/Poisson process as a source for renewal events), which are generally not the case in healthy physiological systems. As a result, incumbent paradigms will not optimally reduce the uncertainty with elucidating/interpreting real-time dynamics captured by raw PTS data.

**Scientific Context—Wiener Hypothesis (WH):** Information flow is a real-world process that can drive the dynamics of a system. This flow is measurable [2,51], and all signal processing should explicitly account for the WH. Nested systems automatically entail multiple spatial and temporal scales in system dynamics. The extension of the WH across the entire CCC offers a paradigm to understand how these nested processes interact and, moreover, reinforce the relevance of fractal signal processing.

The supporting material (SM3) presents a way to preform fractal-signal processing on raw PTS data via modified DEA (MDEA). This method can extract the time-varying scaling parameter from a PTS, making fractal signal processing tangible in the context of practical workflows. The workflow is presented as a “recipe” that can be immediately be used as a conceptual scaffold by the user to process PTS data (or any dataset with nested processes).

### 2.4. Fractal Time Entails MFD Synchronization

A key aspect of the body is that each of its nodes is a nested network. For example, intra-cellular networks of nucleic acid communication give rise to intra-cellular protein networks, which ultimately can give rise to inter-cellular communication networks, ultimately giving rise ONs and to NoONs. Consequentially, each of these networks in the hierarchy communicates on a different time scale. For instance, intra-cellular networks will likely transmit signals over a more rapid timeframe compared to inter-cellular networks. Thus, a given measurable physiological response is the result of many networks coordinating their actions across multiple different time scales.

This massive coordination requires that our notion of time be reframed to account for these multiple-scale coordinations. The MFD of each ON time series is a measure of the time-dependent complexity of that ON time series, and it is the matching of the MFD ON time series that provides the synchronization referred to as CS [66]. We call this more precise definition *fractal time*.

**MFDs and health**: Each communication strategy within a given network can be identified as a temporal fractal. Given that an NoON is comprised of a large number of nested ONs across different time scales, an ON time series can be associated with an MFD time series (each MFD is related to the communication strategy of an ON contributing to the overall nested NoONs). MFDs imply that the ON has a greater set of options available when it needs to respond or adapt to a new variation in the environment than do a mono-fractal time series.

In the context of the FAH, we further hypothesize that a targeted clinical intervention which increases its MFD—that is, restores the option set that a patient’s given ON has in health—has therapeutic potential. However, this hypothesis has yet to be rigorously tested in a clinical setting where diminished MFD implies a reduction in this option set, which corresponds to disease or injury. West et al. [46] collected the empirical evidence to support the related CS hypothesis (CSH) in rehabilitating injured or diseased ONs, with which they close their book as follows [46]:

**CSH**: An injured or diseased ON can be rehabilitated to a healthy level of functionality using a CS-protocol. The CS-protocol is to systematically drive the compromised ON by a second, real or simulated, sender-ON signal having the healthy fractal dimension properties of the receiver-ON being rehabilitated. The CS-protocol minimizes the time to re-establish a spontaneous self-generating state of health in the compromised ON.

**Information flow builds “software” to drive physiology:** Certain key mathematical concepts that relate to the dynamics of information flow are the notion of ergodicity and nonergodicity. However, in a practical sense, it will be helpful to reframe ergodicity as “(relatively) *Information Poor*” and nonergodic as “(relatively) *Information Rich*”. Information follows a gradient on which it flows from an information-rich ON to a relatively information-poor ON, and identifying the gradient of information flow is possible through leveraging the methods of fractal analysis of physiological time series signals that arise from inter-ON communication [17]. This can be accomplished by applying the algorithms of *DEA and MDEA* to the time series (see SM3 for details).

### 2.5. Network Medicine and FAH Entailment

The discipline of *Network Medicine* has in large measure developed around the technical entailments of ’fractal time’ in physiology. This stems from the realization that the FAH has remarkable entailments, including that EEG time series have fractal statistics as a necessary requirement to satisfy the *Principle of Complexity Matching and Management* [11,17,21] to optimize the efficient information exchange among ONs (e.g., among EEG channels). These channels receive information from various sensors which they process, ultimately sending messages (processed information) to multiple receivers to carry out instructions [17]. However, the human brain is not just a passive receiver of sensory information nor is it just a control-ON for the body, but it is self-aware, being cognizant of its own creativity using memory, reasoning, etc., and of its ongoing changing internal behavior in response to changes in the external environment.

The body’s communication network can only be optimal if each ON in the NoONs which is the body itself that can generate, integrate, and transmit MFD time series in a way that is compatible with the PCM&M. Also note that FAH provides a generic strategy for testing our understanding of the operation of the human brain not unlike what Per Bak did for our understanding of the behavior of many-degree-of-freedom systems governed by the emergent dynamics of spontaneous self-organized criticality (SOC) [60]. Thus, the next step toward understanding the spontaneous self-organization of living systems is that in living ONs, the scaling behaviors arise from emergent processes described by spontaneous self-organized temporal criticality (SOTC) [16,97]. Criticality is the dynamic condition that gives rise to the onset of phase transitions; in its simplest form it is generated by a control parameter adopting a critical value which changes the behavior of the intra-ON dynamics from being short-range to long-range [98]. Thus, the CETS with MFD are generated.

In a Network Medicine context, the FAH provides a degree of generality by means of the ON-generated MFD time series entailing an information force within a NoON. The idea of an information force follows from that of a thermodynamic entropic force which in the present context involves information flow manifest by an MFD time series [99] entailed by the principle of MFD synchronization (PMFDS) that suggests the existence of an information force created by the information field generated within NoONs. The PMFDS entails that the CETS carries its crucial information via a time-dependent scaling index $\delta_{j}\left(t\right)$ generated by ${\mathrm{ON}}_{j}$ and has the following properties [2,97] (the various indices and their relations with one another are recorded in Figure 1):

(1) The time series is composed of discrete events that are statistically independent of one another and are therefore renewal events (REs).
(2) The time intervals between successive REs are described by an IPL PDF ($\tau^{-\mu }$) and therefore constitute CETS.
(3) The complexity of the CETS X(t) is measured by the MFD scaling index δ of the scaling PDF in phase to be $P\left(x,t\right)=1/t^{\delta \left(t\right)}F\left(x/t^{\delta \left(t\right)}\right)$ (see SM1 for details involving the FOC).
(4) The MFD is determined by the complexity of the time series in property 2 such that the MFD is equal to the IPL index D(t)=μ(t), and the IPL index is related to the scaling index in property 3 by μ(t)=2−δ(t) (see SM1 for details).

**Compliance with Natural Law:** How has Nature elected to efficiently handle the information entering, being utilized, leaving, or being stored within NoONs such as in the mammalian brain? This question was answered in part by the PMFDS, which requires that each information bearing ON-generated time series has a time-dependent fractal dimensionality that measures its changing complexity level. At the interface of two such ONs, it was empirically determined by West et al. [2] and Mahmoodi et al. [1,51] using DEA processing of the measured time series that information is exchanged between the two ONs following the information gradient in accord with the PCM within a human, which is a NoON [17], between two humans walking [21] or talking [19] or even between an individual and a social group [10].

## 3. Formal Properties of CETS

It is important to understand the dynamic origin of CETS as well as the significant role they play in the transport of information from one complex network to another [17]. CETS are a manifestation of cooperative interactions between the units of a complex dynamic network that can spontaneously reorganize itself after being disruptively perturbed and is usually referred to as self-organized criticality (SOC). We have come a long way in understanding SOC, since the original work of Bak et al. [60] over a quarter century ago, including a new approach to SOC that emphasizes the temporal over the intensity anomaly PDF [16,61]. Mahmoodi et al. [16] defined this manifestation of spontaneous self-organization as self-organized temporal criticality (SOTC). According to SOTC, the CEs just defined are the events that Allegrini et al. [62] found in heart beats leading to the quantitative method to distinguish healthy from pathological subjects. This method was subsequently related to the MFD of the heartbeat time series [91].

### 3.1. Generating CETS

So, how do networks generate these CEs, and once generated, how do other networks detect them when they are intermixed with many non-CEs with the same form of waiting time PDF? Let us consider the ostensibly simple dynamic model following in part from [11,56]. A particle moves in the interval I≡[0,1] with a trajectory X(t) governed by the following equation:

(7) $$\frac{dX(t)}{dt}=aX{\left(t\right)}^{z}\;,\;z>1.$$

This dynamic equation serves the purpose of generating non-Poisson renewal statistics when the particle’s trajectory intercepts the boundary at X(t)=1 and the particle is reinserted at a random point within the interval *I*. In other words, when the particle reaches the border X=1, the clock is reset, and the particle is injected back to a new initial condition 1>X(0)>0, with uniform probability. Each reinsertion constitutes a CE, and the sequence of reinsertions constitute a time series of CEs, which is a pure CETS.

Imagine that each sojourn time within the interval *I* is recorded via direct observation, and for clarity, assume these random events to be visible. In this way, we obtain for the time intervals between successive CEs through the series $\left\{\tau \right\}\equiv \tau_{1}$, $\tau_{2}$,···, where $\tau_{j}$$=t_{j}-t_{j-1}$, and $t_{j}$ is the time of the $j^{th}$ reinsertion, as in our dataset. Integrating Equation (7) yields the analytic relation between the sojourn time and the random value y=X(0) initiating that sojourn:

(8) $$\tau =\frac{1}{a\left(1-z\right)}\left[1-y^{1-z}\right].$$

The sojourn or waiting time PDF ψ(τ) and the PDF p(y) for the initial state are consequently related by ψ(τ)dτ=p(y)dy, since the probability of having a given random initial condition is the same as having the associated random sojourn time given by Equation (8). In the case of a uniform PDF of reinjection points p(y)=1, after some algebra, we obtain the hyperbolic form for the waiting time PDF:

(9) $$\psi \left(\tau \right)=\frac{\left(\mu -1\right)T^{\mu -1}}{{\left(T+\tau \right)}^{\mu }},$$

where τ>>T asymptotically becomes an IPL. In terms of the original parameters, the IPL index μ is μ=z/(z−1), and a characteristic time is T=1/[a(z−1)] of the waiting time PDF. The probability that a CE has not occurred up to a time *t* is defined as follows:

(10) $$\Psi \left(t\right)=\underset{t}{\int^{\infty }}d\tau \psi \left(\tau \right)={\left(\frac{T}{t+T}\right)}^{\mu -1},$$

and is the survival probability.

The average time between CEs obtained using the hyperbolic PDF is defined as follows:

(11) $$\langle \tau \rangle \equiv \underset{0}{\int^{\infty }}\tau \psi \left(\tau \right)d\tau =\left\{\begin{array}{c} \frac{T}{\mu -2}\;\;\mathrm{for}\;\;3>\mu >2\\ \infty \;\;\;\;\;\mathrm{for}\;\;2>\mu >1 \end{array}\right..$$

We have defined a CETS as a renewal point process with a waiting time PDF that is an IPL in the time intervals between events. With this in mind, recall the discussion of the information transfer properties using the memory associated with renewal dynamic process discussed by Feller [57] for the infinite memory case for a nonergodic process defined as having μ<2. But this renewal generated memory is not the only form of memory in PTS [62].

### 3.2. The Wiener Hypothesis

In a popular 1948 lecture, which is to say a lecture containing no mathematical equations, Wiener observed [100] that according to thermodynamics, two complex networks, under normal conditions—one high in energy (hot) and another low in energy (cold)—when brought in contact with one another transfer energy from the hot to the cold network. This is, of course, the second law of thermodynamics in which it is implicitly assumed that both networks are high in entropy. The second law is therefore predicated on the network’s dynamics being energy-dominated in which the control of behavior is determined by the network with the greater free energy.

Wiener goes on to conjecture that if the hot network is low in information and the cold network is high in information, then information may be transferred from the cold network to the hot network. This directionality of information flow includes the possibility that the hot network can be controlled by the cold one. Schrödinger [101] introduced the term negentropy to quantify how a living being extracts order from the environment and discards disorder. He further explained that it is necessary to locally violate the second law of thermodynamics by exchanging entropy with the environment and in so doing maintain the ordered state of an organism’s life while disrupting its immediate environment.

**Figure 7:**
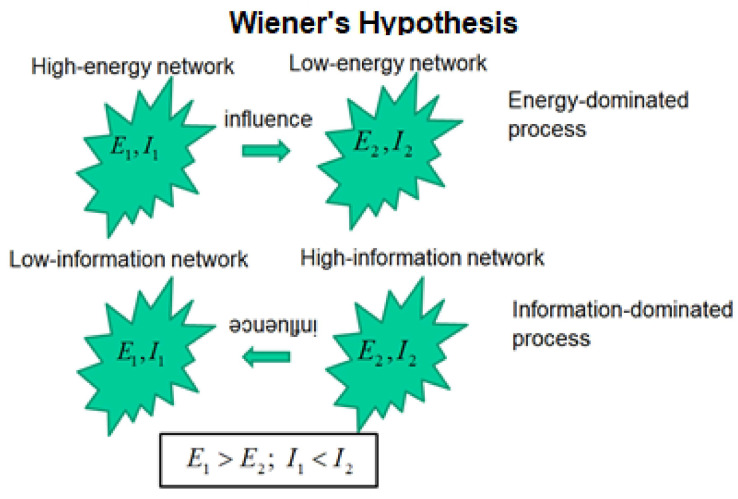
Wiener’s Hypothesis: The upper panel denotes the familiar thermodynamic situation of an energy-dominated interaction. The lower panel depicts the counter-intuitive information dominated interaction. This is emphasized by the influence in the lower panel being turned on its head. Adapted from [17].

He (Wiener) identified information with negentropy and saw these kinds of processes as being information-dominated and assumed that they need not be physical in nature. In such situations, unlike the familiar energy-dominated processes of thermodynamics in which the hot network governs things, what is of interest to us are the information-dominated processes wherein cold networks control the behavior of hot networks, as suggested in Figure 7.

Wiener speculated that the complex networks in the social and life sciences behave differently from, but not in contradiction to, those in the physical sciences, with control emanating from the flow of information and not only from the flow of energy. The significance of this observation cannot be overstated. In the physical world, cars roll down hill, traveling from higher to lower potential energy and fresh-from-the-oven apple pies cool off, with heat radiating from the hot apples to the cooler room. The force laws and therefore control in physical phenomena are a consequence of the negative gradients of an energy potential. Wiener did not explicitly dwell on this point, but his speculation entails that the force laws controlling social phenomena need not follow the negative gradients of energy potentials (even when they can be defined) but instead follow gradients produced by the local imbalance of information.

The story of how information forces come into being is the thread that connects the various aspects of our narrative into a coherent whole. The point of departure is a recasting of Wiener’s observations into the form of a hypothesis that was proven in stages, as recorded in [11], and is called the Wiener Hypothesis:

**WH**: Given the proper conditions, the force between two complex dynamic networks, produced by an energy gradient acting in one direction between the networks, can be overcome by the force produced by an information gradient acting in the opposite direction between the networks.

### 3.3. Information Exchange Between Networks

Networks appear in many shapes and sizes, and not all of them act for our benefit. The physiologic networks controlling the activity of the brain, heart, lungs, legs, *etcetera*; the economic webs of global finance and stock markets; the differing social meshes of governments and groups of terrorists; the physical wicker of the Internet and climate change; the bio-networks of gene regulation and the human body; and the ecowebs of food networks and species diversity all bear a striking similarity. As the networks in which we are immersed become increasingly complex, several universal properties emerge. One of those properties is a version of the WH having to do with how complex networks, perhaps involving phenomena assigned to very different disciplines, exchange information with one another.

The first order of business in establishing the truth of the WH requires that we provide a working definition of complexity. We note empirically that the dynamic behaviors of complex networks are expressed in terms of PSDs, wherein the amount of energy in each frequency interval is recorded. In all the situations to date, complexity arises when the power spectrum $S_{p}\left(f\right)$ takes on an IPL form:

(12) $$S_{p}\left(f\right)\propto \frac{1}{f^{\beta }},$$

with the IPL index β in the empirical interval 1<β<3, which goes by the technical name of 1/f-noise in the older literature or as 1/f-variability in the more modern literature. In fact, 1/f-variability is taken by many scientists to be the signature of complexity and appears in a vast array of dynamic phenomena, including human brain activity [102], body movements [84], music [103,104,105], physiology [55], genomics [106], sociology [107], and many more that are discussed in [11].

**Figure 8:**
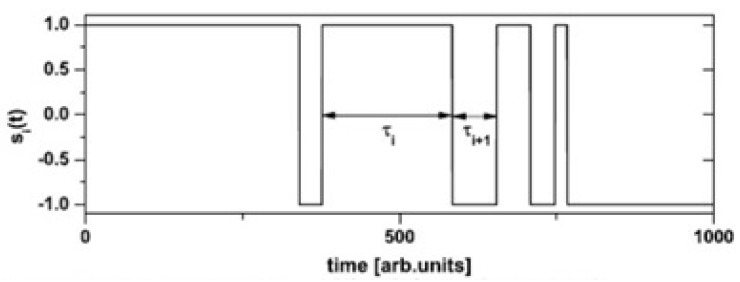
The time series for two-state renewal process is depicted in which the switching time betweeen states is deterimned by an IPL PDF. The time interval between successsive CEs, say between event *j* and j+1, is $\tau_{j}$ as shown.

For establishing the veracity of the WH we adopt 1/f-variability as our working definition of complexity and the IPL index μ as its quantitative measure. We focus on discrete random processes, since these are the more common form of experimental data, particularly the time series recording the occurrence of CEs, e.g., heart beats, stride intervals, earthquakes, solar flare eruptions, starting and stopping of traffic flow, and so on. Such a discrete process $s_{1}\left(\tau \right)$ is depicted in Figure 8, and the events are the transitions between +1 and −1 recorded as the time intervals between successive events $\tau_{1}$,···,τ, as a time series with *N* data points.

In the time domain, a complex network has a waiting time PDF ψ(τ) with a corresponding IPL index given by μ=3−β; see Figure 1 for a discussion of the relations among the scaling indices such that the asymptotic PDF for the time intervals between CE is as follows:

(13) $$\psi \left(\tau \right)\propto \tau^{-\mu }.$$

The quantity $\psi \left(\tau \right)d\tau$ is the probability of the time interval between successive CEs being generated by the network dynamics is in the time interval (τ,τ+dτ); see Figure 8. Consequently, networks characterized by 1/f-variability have a long-time memory whose extent increases with a decreasing IPL index.

## 4. Detecting Empirical CEs in Datasets

**Figure 9:**
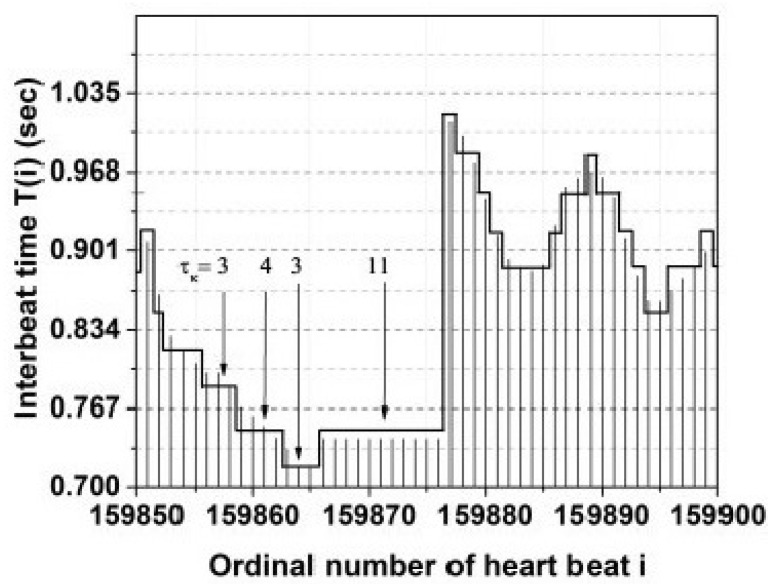
A CE is defined as the experimental curve given by the thick black line crossing the border between consecutive stripes. The symbol $\tau_{\varkappa }$ indicates the time interval in terms of the heartbeat number ϰ between consecutive CEs defined by the black line crossing from one stripe to one of the neighboring stripes. The width of a single strip is ΔT=1/30 s. From [91] with permission.

Bohara et al. [91] point out that the detection of CEs is the major problem encountered in proving that SOTC is the process driving the empirical phenomenon under study, whether that phenomenon is in the physical, social, or medical domains. The approach adopted in detecting the often-invisible CEs in HRV time series is sketched in Figure 9, wherein the experimental signal is obtained by assigning to each heartbeat a value corresponding to the time interval between successive beats. The interbeat time (horizontal) axis in the figure is divided into narrow stripes of size ΔT which define the occurrence of an event as the experimental signal crossing from one stripe to one of its two nearest neighbor stripes. The heartbeat trajectory may remain in a given strip for an extended time interval, suggesting the typical fractal intermittency behavior that led to the discovery of CEs in the first place. However, the experimental signal crossing the border between contiguous stripes is not necessarily a CE. We call this use of stripes in connection with the DEA RW a modified DEA (MDEA).

The CEs are renewal, and consequently, the times $\tau_{j}$ should be uncorrelated. The breakdown of the renewal condition is assessed by evaluating the time-average correlation function C(t), where the time average is indicated by an overbar:

(14) $$C\left(t\right)=\sum_{\left|t_{i}-t_{j}\right|=t}\bar{\frac{\xi \left(t_{j}\right)\xi \left(t_{j}\right)}{\sigma^{2}}},$$

and σ is the standard deviation of the time series. The empirical time series $\xi \left(t\right)$ is thought to consist of a combination of CE and non-CE as described in detail in Section 5.3. Consequently, C(0)=1 and for a genuine CETS, we have C(t)=0 for t>0. But on the contrary, we find for one time step that the auto-correltaion function yields C(1)≈$\epsilon^{2}$, where ϵ is defined to be the probability that an event is a CE; see Equation (18) for a theoretical description of such a mixture of events. The square of ϵ indicates the probability of both events in the correlation function are CEs.

### 4.1. Heart Rate Variability (HRV)

Normal sinus rhythm is an over-simplification of the rhythmic pattern made by the series of time intervals between successive human heartbeats [64]. This phrase implies that heart beats are steady and regular, with relatively little variability. But this is not what is observed; in fact, the frequency spectrum depicting heart rate variability (HRV) is asymptotically IPL and fit by Peng et al. [64] to a truncated Lévy process.

Tuladhar et al. [108] show that HRV time series are closely tied to CEs through their statistical scaling behavior. They have shown that CEs play a fundamental role in the transport of information between complex networks. The majority of events under observation in the case of HRV time series [62,91] are not CEs, which are rare, and are imbedded in a sea of pseudo-CEs. Making these invisible CEs visible is accomplished by converting the HRV time series into a diffusion process described by the scaling PDF given by Equation (17). To reiterate, for classical diffusion, the scaling index has the value δ=0.5, and the function F(·) has a Gaussian form. Anomalous diffusion is measured by how far the scaling index deviates from its classical value, which can be measured using the WS information entropy.

**Figure 10:**
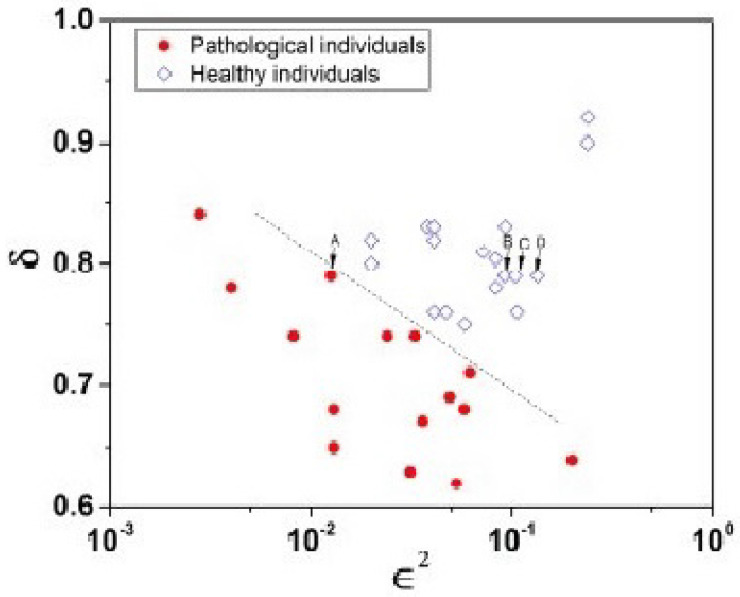
Using the scaling index and the probability that the event is a CE to distinguish between subjects that are healthy (above diagonal dotted line) from those with pathological HRV (below diagonal dotted line). From [91] with permission.

The electrocardiogram (ECG) records of the *MIT-BIH Normal Sinus Rhythm Database and of the BIDMC Congestive Heart Failure Database* for healthy and congestive heart failure patients, respectively, were used in the application of MDEA and the correlation function. For each subject, both δ and $\epsilon^{2}$ were calculated, and the results depicted in Figure 10 turned out exactly the same in both [62] and [91]. Values of $\epsilon^{2}$ larger than 0.05, ϵ>0.22 are referred to as weak randomness, and values of $\epsilon^{2}$ smaller than 0.05 are referred to as strong randomness. But this distinction must be used with caution because, as is evident in the figure, the distinction between healthy and pathological individuals requires knowledge of the scaling index δ as well as that of $\epsilon^{2}.$ Thus, a criterion has been established to distinguish patients with pathological from those with healthy HRV time series based on CEs.

We notice that the ideally healthy condition would correspond to δ=ϵ=1. This condition means that an ideal sequence of CEs does not host any pseudo-CEs events and would have μ=2, which is the border between the region of perennial aging, μ<2, and the region where the rate of randomness production becomes constant in the long-time limit, μ>2 [17]. A patient’s HRV time series moves toward the pathological condition as their scaling becomes closer to the scaling of ordinary diffusion δ=0.5, namely, closer to the border between the region of CEs (2<μ<3) and the Gaussian basin of attraction (μ=3).

In Figure 10, we have indicated four patients denoted by capital letters: the first patient is denoted A and is interpreted to be unhealthy, since this patient is below the indicated diagonal, whereas the three other patients B, C, and D are above the diagonal and are interpreted as being healthy. Patient D is further above the diagonal than the other two patients and is therefore healtier than C, who is healthier than B. This is not an arbitrary assignment of health values. This ordering of a patient’s health is shown in Figure 6 to be consistent with the average MFD of a patient’s ECG time series.

In Figure 6, the HRV MFD time series (MFDTS) for each of the four patients is processed to yield the symmetric fractal dimension spectra and which peaks at the average fractal dimension of the patient’s MFDTS. The interpretation of the left to right ordering in Figure 10 as being indicative of the patients’ relative states of health consistent with the ordering of the average MFDs depicted in Figure 6. The fractal dimension is our measure of complexity which in turn is our the measure of health [68], and the two orderings are consistent. In addition, the width of the MFD spectra provides an independent measure of health so that the widths of the spectra in Figure 6 indicate an ordering from the narrowest to the boadest yielding A, B, C, and D, thereby supporting the interpretation of the relative health of the four patients. This argument provides two independent measures of the health of patients for the same scaling index.

### 4.2. Electroencephalograms (EEGs)

Brain dynamics research has disclosed the existence of CEs and shown that the existence of CEs is responsible for the 1/f-variability in brain wave data [1,2,51]. Perhaps of equal importance is establishing that although CEs are generated by critical dynamics, they remain compatible with the wave-like nature of brain processes. Bohara et al. [109] showed that although criticality generates large deviations from the regular wave-like behavior, brain dynamics also host CEs in regions of nearly coherent oscillations, thereby making many CEs virtually invisible. Furthermore, the anomalous scaling generated by the CEs was established with high accuracy by means of DEA of raw data, which is suggested by the theoretical perspective not requiring the CEs to yield a visible physical effect (see SM3 for details).

Bohara et al. [109] obtained three main results: (1). Confirmation of the critical role of CEs in brain activity. (2). Demonstration of the theoretical tools necessary to understand the joint action of CEs and periodicity. (3). Shed light on the nature of the central role of CEs in the spontaneous dynamic self-organization of the brain and thereby contributed to our understanding of cognition.

Jelinek et al. [94] used the fact that the complexity of cognitive tasks is associated with the mental effort required to address a difficult problem, thereby leading to an increase in the IPL index with increasing task difficulty to examine the conjecture that the evolution of disease leads to μ moving from the ideal healthy condition μ=2 toward the border with Gaussian statistics with μ=3; as the disease worsened, they examined HRV time series of patients affected by diabetes-induced autonomic neuropathy of varying severity and determined that the progression of cardiac autonomic neuropathy (CAN) does indeed shift from 2, the border with perennial variability, to 3, the border with Gaussian statistics. This index thereby provides a new and sensitive index for measuring disease progression. At the Gaussian border, the complexity of the CE’s time series dynamics simplifies to FBM, that is, to Gaussian fluctuations with long-term memory.

There is general agreement concerning the importance of 1/f-variability for neurophysiological processes, and the spectrum of 1/f-variability can be realized in two distinct ways. Type I 1/f-variability has an FBM explanation first articulated by Mandelbrot [28,78]. This is distinct from Type II 1/f-variability generated by CEs that was first identified by Allegrini et al. [62]. These separate sources of 1/f-variability have been confused in the literature because their generating mechanisms can appear separately or together depending on the level of complexity of the phenomenon under investigation.

**Figure 11:**
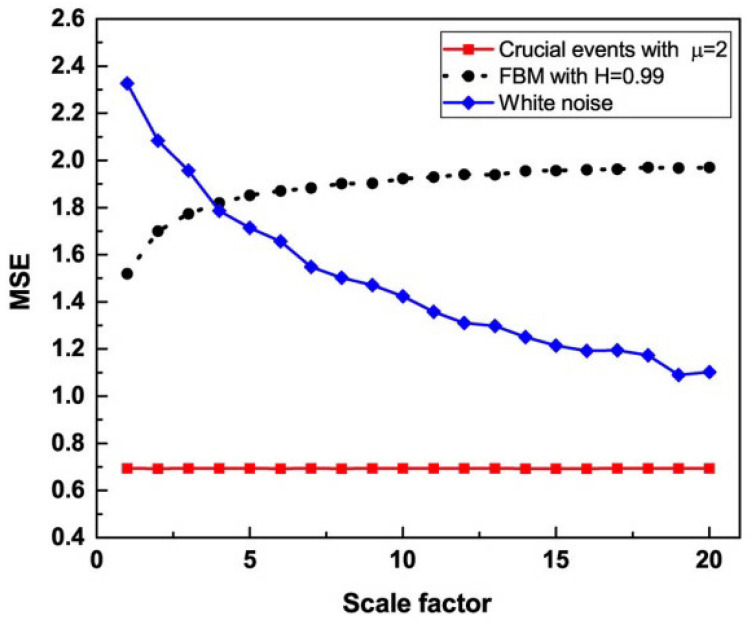
The multiscale entropy (MSE) is graphed vs. an aggregation scale factor. Although both the FBM and that with CEs time series generate 1/f-noise, the trajectory with CEs is independent of the size of the scale factor, while the FBM trajectory is only asymptotically independent of the size of the scale factor. Finally, white noise depends strongly on the scale factor. From [94] with permission.

Figure 11 depicts multiscale entropy (MSE) processing of both Type I and Type II 1/f-variability, which have confused researchers studying HRV and the time series generated by other complex networks. The black line in the figure illustrates Type I 1/f-variability that asymptotically agrees with the traditional FBM 1/f-variability. The red line describes Type II 1/f-variability generated by CEs and is significantly different from the traditional expectation of 1/f-variability. Both types of 1/f-variability differ significantly from the blue line depicting white noise. The analysis presented by Jelinek et al. [94] established that RR fluctuations host both forms of 1/f-variability and that in the *Definite* CAN time series, only Type II 1/f-variability remains. This remarkable conclusion was reached through the proper use of Rényi entropy.

As we remarked earlier, the condition μ = 2 is the ideal healthy condition. However, the heartbeat process hosts not only CEs but also non-CEs that can be either ordinary Poisson events or events generated by Type I 1/f-variability. It is important to stress the existence of this kind of non-CE because prior to the result of the analysis by Jelinek et al., the Definite CAN patients were still categorized by 1/f-variability. The non-CEs generated by Type I 1/f-variability contribute to increasing the concentration of non-CEs given by 1−ϵ. When ϵ = 0, all the events are non-CEs and are a combination of ordinary Poisson events and Type I 1/f-variability events. When ϵ = 1, all events are CEs. Between these extremes where ϵ < 1, there does not yet exist a way to count the relative number of the event types.

**Figure 12:**
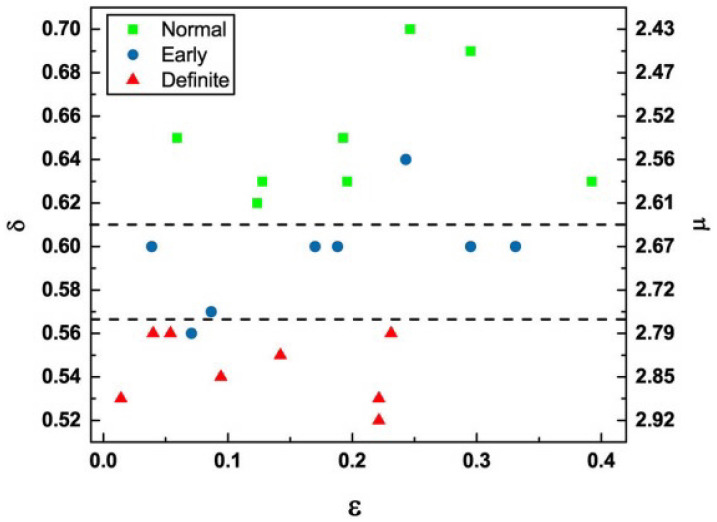
DEA scaling index on left (vertical-axis); MFD index on right (vertical-axis) versus the correlation rate (horizontal-axis) of the HRV time series for participants in different stages of CAN. From [94] with permission.

In the absence of a counting technique, the authors in [94] applied the MSE to show that the heartbeat process under observation is not white noise but is 1/f-variability. The fact that increasing the values of the IPL index μ is beneficial is established in Figure 12, which shows that *Definite* CAN patients are characterized by small values of ϵ, while *Normal* CAN patients seem to move toward the condition of larger values of ϵ.

Figure 12 depicts the results obtained from patients with varying severity levels of CAN. The division into three groups by the two dashed lines is to some extent arbitrary. However, the Definite patients tend to have small values of ϵ and the scaling parameter values δ are closer to the border with ordinary diffusion, i.e., δ = 0.5. There is only one Early patient with δ in the Normal region and only one other Early patient in the Definite region. The results in this figure provide strong support for the hypothesis that CEs are an important signature of healthy physiological function. Moreover, either an excess of non-CEs (smaller ϵ) or transition from the healthy condition of μ close to 2 to values close to 3 and beyond is a important signature of disease progression.

## 5. Complexity-Entailed Principles

We have restricted our discussion to complex networks that can be quantified by IPL PDFs, and consequently, the IPL index μ has been used to quantify our working notion of complexity. Using this concept of complexity, we can determine how information is exchanged between two such networks, or in a more limited sense, how information is transferred from one complex network to another complex network and use this to support the WH. This hypothesis remained a provocative speculation for over sixty years. It was only with the relatively recent activity to develop a science of networks that an extended form of the WH was proven to be true.

### 5.1. Principle of Complexity Matching and Management

As a physicist, the natural first approach to proving the WH was to apply linear response theory (LRT) to the perturbation of one complex network by another. However, we immediately encountered the fact that the response of nonstationary networks to harmonic perturbations had been found by a number of significant researchers [110,111,112,113,114,115,116] to fade away with time, which is an effect called the *death of LRT* by Sokolov et al. [116] and others. However, our group was able to establish that the demise of LRT was grossly exaggerated. Aquino et al. [117] showed that it is possible to go beyond the “death of linear response” and establish an asymptotic correlation between an external stimulus and the response of a complex network generating nonergodic renewal processes by taking as stimulus a similar nonergodic process. Subsequently, the same research quartet [18] implemented a generalized fluctuation–dissipation theorem to show that the ideal condition of 1/f-variability for two interacting networks corresponds to maximal information transport [17].

The proof of the WH consequently relies on generalizing some of the fundamental ideas of nonequilibrium statistical physics, in particular generalizing the linear response theory (LRT) to include out-of-equilibrium, nonstationary phenomena [18,117] which we write as follows:

(15) $$\langle \xi_{S}\left(t\right)\rangle =\overset{t}{\int_{0}}\chi \left(t,t^{\prime }\right)\xi_{P}\left(t^{\prime }\right)dt^{\prime },$$

where the brackets here denote an average over the network response to a Gibbs ensemble of perturbations. In this general form of the LRT, both the time series from the perturbing network $\xi_{P}\left(t\right)$ and from the responding network $\xi_{S}\left(t\right)$ consist of intermittent CEs. In the stationary case, this LRT becomes indistinguishable from the traditional prediction [118], as it should, but in the nonstationary and nonergodic case, it is significantly different. The analysis revolves around the properties of the linear response function $\chi (t,t^{\prime })$, which is the time derivative of the auto-correlation function of the responding network. The details of what the generalization of LRT entails what is given by West and Grigolini [11], and we now turn to a discussion of the results which are valid for the asymptotic cross-correlation function evaluated as an ensemble average [18] and as a time average [119].

**Cross-correlation cube (CCC):** Consider two complex networks which through their interactions exchange information. Familiar examples include two people talking with one another; a patient’s body ‘talking’ to a physician during a physical examination; and the music of a symphony orchestra exciting an audience member’s brain. Each complex network has its own characteristic exponent, and the efficiency of the information transfer is determined by the relative values of the IPL indices of the sender and receiver [17]. This is an important point, since it is in direct opposition to an explicit assumption made by Shannon in his requirement that information transfer ought to be independent of the properties of the sender and receiver [13]. Shannon was more interested in the engineering properties of the message being transmitted through a channel than he was in the properties of individuals at either end of the message transfer process.

One measure of the efficiency of information transfer between two complex networks is the cross-correlation between the output of a complex network P (perturbing network) and the stimulation of a complex network S (responding network) being perturbed by P. A CETS is generated by an IPL PDF for the waiting time between events and is denoted by ξ(t); see Figure 8. The normalized output of network P is denoted by $\xi_{P}\left(t\right)$, that of network S by $\xi_{S}\left(t\right)$, and the averages over the time series by an overbar; the cross-correlation function is defined as $C\left(t\right)=E[\xi_{S}\left(t\right)\xi_{P}\left(t\right)]$. The notation E[·] is introduced for the average because the results discussed here are proven for both the ensemble average [18] and time average [119] by explicit calculation. The t→∞ cross-correlation function is the simplest measure of the asymptotic information transfer efficiency from network P to S.

In keeping with our working definition of complexity, the networks of interest are complex and have μ<3. For the moment, we focus our attention on networks whose complexity is high corresponding to *non-ergodic region* with 1<μ<2 and networks whose complexity is intermediate corresponding to *ergodic region*, with 2<μ<3. Note that a network in this latter region can make an asymptotic transition from CEs to FBM. But in this section, we restrict our remarks to complex networks generating only CEs. Summarizing the asymptotic influence of one region on another, we show below the following:

(1) A complex network belonging to ergodic region cannot exert any influence asymptotically on a second complex network belonging to non-ergodic region.
(2) A complex network belonging to ergodic region exerts varying degrees of influence on a second complex network belonging to ergodic region. This follows from the PCM.
(3) A complex network belonging to no-ergodic region exerts varying degrees of influence on a second complex network belonging to no-ergodic region. This follows from the PCM.
(4) A complex network belonging to non-ergodic region transmits its full complexity to a second complex network belong to ergodic region, which was anticipated by the WH.

**Figure 13:**
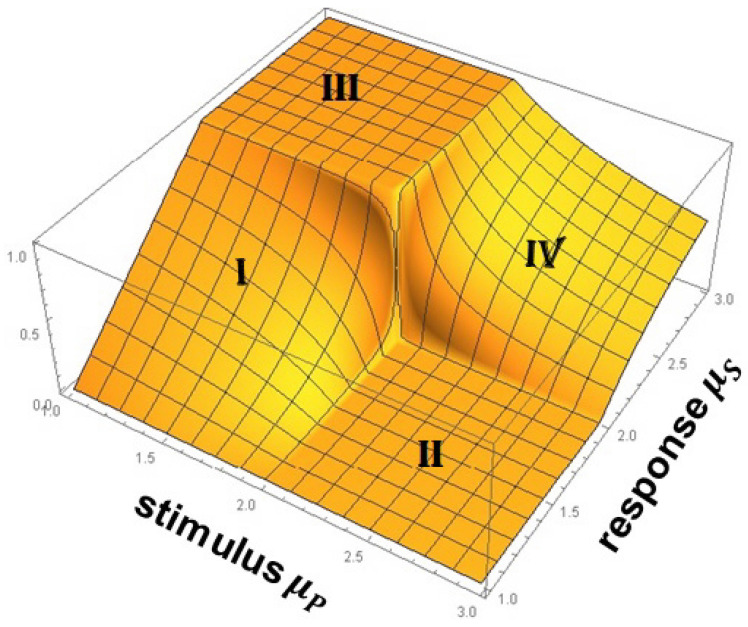
The cross-crrelation cube (CCC) depicts the asymptotic response of the cross-correlation function graphed as a function of the IPL indices of the responding network S and the stimulating network P. The height of the CCC, that being the vertical axis perpendicular to the $\left(\mu_{S},\mu_{P}\right)$—plane, is normalized to a maximum value of one. Adapted from [11] with permission.

In Figure 13, the asymptotic cross-correlation function is normalized to unity and graphed as a function of the IPL indices of the two networks to form a cross-correlation cube (CCC). For a given level of complexity of the stimulating network P denoted by the IPL index $\mu_{P}$ the response of network S depends on its own level of complexity denoted by the IPL index $\mu_{S}$. The values of the two IPL indices define a plane ($\mu_{S},\mu_{P}$), and the value of the cross-correlation function at each point on this plane defines a third dimension so that the three values together give rise to the CCC. Note that this cube denotes the asymptotic values of the normalized cross-correlation function and displays several remarkable properties; see [11] for a complete discussion.

When the two IPL indices are equal to 2, there is an abrupt jump up from 0 (region II) to 1 (region III) or down from 1 to 0, depending on the values of the IPL indices just before they converge on 2. In region III, perfect correlation is realized between the CE dynamics of the two networks. The point $\mu_{P}=\mu_{S}=2$ is a singular point where the spectra of the two networks display exact 1/f-noise.

Note that the interactions between the two networks considered here is uni-directional. This same kind of perturbative interaction under a variety of circumstances is considered below providing evidence that the network with the greater information, i.e., is the more complex network, determines the direction in which information flows, that being the direction of the information gradient, i.e., the *Information Force* (IF) [99], thereby supporting the existence of the PCM&M. These calculations provide insight into the empirical phenomena of habituation examples which are the next topic of discussion. But for the moment, let us return to what more we can learn from the CCC.

The upper plateau of the CCC indicates that when the perturbing network P is nonergodic as $1<\mu_{P}<2$, the average time between CE stimuli ${\langle t\rangle }_{P}$ diverges to infinity. The responding network S is ergodic $2<\mu_{S}<3$, and the average response time separating CE events ${\langle t\rangle }_{S}$ is finite. The typical time intervals between stimulating P-CEs are much longer than those between unperturbed S-CEs. Consequently, the S-CEs have more than enough time to adjust to a given P-excitation, that is, to transfer the influence of the perturbation throughout the S-network before the next perturbation occurs. In this way, the S-network relaxes to the P-network perturbation. The greater information in the nonergodic output of the P-network $I_{p}$ dominates the ergodic statistics of the S-network having information $I_{s}$ and produces complete asymptotic correlation between stimulus and response. The mathematics establishing this result are detailed in [18,119].

The lower plateau of the CCC indicates that when P is ergodic $2<\mu_{P}<3$, the average time between P-CE stimuli ${\langle t\rangle }_{p}$ is finite, and when S is nonergodic $1<\mu_{S}<2$, the average S-CE response time ${\langle t\rangle }_{s}$ diverges. The typical time intervals between P-CEs are therefore much shorter than those naturally occurring between the S-CEs. The responding S-CEs are much less numerous than those of the stimuli, which interfere with one another, and their influence is lost before the responding network has time to transfer the perturbation even a short distance within its network. Consequently, there is no detectable response asymptotically. The information-rich S network, $I_{s}>I_{p}$, is seen to be unresponsive to the influence of the stimuli due to the significant difference in time scales.

In the two regions in which the IPL indices are in the same domain $1<(\mu_{S},\mu_{P})<2$ and $2<(\mu_{S},\mu_{P})<3$ result in both mean times being either divergent or finite together. The information content of the stimulus and response behaviors are consequently similar so that the value of the cross-correlation function depends in detail on their respective values, as seen in the figure. The analytic expressions for the size of the cross-correlation in these two regions are discussed in [11].

We conclude from studying the CCC that the manner in which one complex network responds to perturbation by another complex network is determined by which of the two networks has the greater information according to the statistics of their respective dynamics. The WH is described by the influence of the stimulus, as it appears on the upper plateau region of the CCC where the information in the stimulus exceeds that in the response. In all regions except the lowest one, a weak stimulus significantly modifies the properties of the responding network. In the upper plateau region, the stimulus not only influences but actually dominates the asymptotic properties of the response and reorganizes them, just as Wiener speculated. The PCM is embodied in the CCC that incorporates the WH into this larger principle.

**Habituation:** Let us now consider the phenomenon of habituation as a categorical exemplar of the information transfer from one complex network to another. This empirical transfer can be explained by means of the PCM&M using the CCC. Habituation is a simple yet ubiquitous form of learning through which animals, including humans, learn to disregard stimuli that are no longer novel, thereby allowing them to attend to new and perhaps more important stimuli [120].

One of the more interesting aspects of habituation is that it can occur at different levels of the nervous system. For example, with strong odors, sensory networks stop sending signals to the brain in response to repeated exposure to the olfactory stimuli. But odor habituation has been shown in rats to also take place within the brain, not just at the sensory level. The statistical habituation model (SHM) [9] hypothesized that 1/f-variability, characteristic of complex networks and arising as it does in both single neurons and in large collections of neurons, is the common element that explains suppressing signals being transmitted to the brain and inhibiting signals being transferred within the brain.

**Figure 14:**
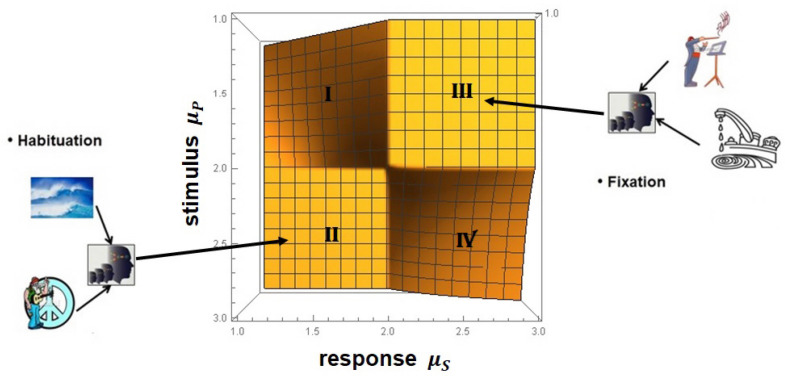
The CCC depicts the asymptotic response of the cross-correlation function graphed as a function of the two IPL indices of the responding network S and the stimulating network P. This is the view of the CCC from above. Examples of stimuli that habituate asymptotically in region II and those that are in ‘1/f-resonance’ with the complexity of the human brain and consequently fixate in region III, like a melody you cannot get out of your head. From [11] with permission.

Repetitious stimuli of unvarying amplitude and constant frequency content, such as a strong odor, a persistent hum, or the gentles sway of a boat, all induce responses that fade over time even though the stimulus persists, since no new information is being presented.This is the situation captured by the region with the greater information $I_{s}>I_{p},$ or region II in Figure 14. The habituation response to the lack of new information allows the brain to shift its focus from the more to the less familiar, with the latter providing new information that may have value an individual may use to act in their self-interest, such as getting a good night’s sleep.

Consider the case of waves crashing on a beach and their sound coming in through a window of a vacation motel at night after a long day of reading on the beach. This naturally generated sound is typically a broadband spectrum of uncorrelated frequencies with random amplitudes and phases. Most people habituate to this pleasant auditory experience and after a short time, they no longer hear it and fall asleep. The hippie playing the guitar in Figure 14, on he other hand, is meant to represent simple, or uncomplicated, music, such as a ballad, which would facilitate rather than disrupt the onset of sleep. In the present context, it is possible to prove using SHM that the response of the brain to such stimuli fades as the IPL in time $1/t^{2-\mu }$ [9].

The plateau region II in Figure 14 is the parameter region $2<\mu_{P}<3$ and $1<\mu_{S}<2$ where the dynamics of the receiving brain and the external stimulus asymptotically become independent of one another. Brain activity in the nonergodic regime is asymptotically unresponsive to ergodic and/or periodic stimuli; the complexity of the neuron network essentially swallows up simple signals through its complex dynamic interactions, and the response fades in time, as described by an IPL.

The asymptotic suppression of periodic stimulation of a complex physical network using linear response theory (LRT) was previously demonstrated by a number of investigators [114,115], which they separately used to argue for the demise of LRT. In the present context, a generalized LRT was used to determine the asymptotic suppression of the stimulus to explain the phenomenon of habituation [9].

However, we know the brain does not habituate to all external stimuli, so let us consider two distinct kinds of stimulation: one ergodic $2<\mu_{P}<3$ and another nonergodic $1<\mu_{P}<2$. The ergodic perturbation can be expressed as a simple spectrum, which allows us to generalize a previously established result for a periodic stimulation of a complex network [114,115]. The nonergodic stimulus can be drawn from a number of sources; here, we chose for contrast the sequence of splashes from a dripping faucet [121] and certain pieces of classical music [105]. The sequence of sounds generated by the water from a leaky faucet splashing into a sink can set your teeth on edge and lead some to toss and turn throughout the night. The statistics of the leaky faucet stimulus was empirically determined to have a PDF with an IPL index in the domain $1<\mu_{P}<2$ and consequently entailed statistics of the time intervals between splashes to be nonergodic [122]. The brain’s response to this ongoing perturbation ramped up from 0 to 1 as the index $\mu_{S}$ increased from 1 to 2, as shown in region I of Figure 14. Over the interval $2<\mu_{S}<3$, the response to the intermittent splashes given by the CCC was maximal. The high plateau III depicts the parameter domain where the brain is ergodic and records the sound of every intermittent drop of water, just as Wiener anticipated in his speculation.

Of course, it is not just annoying stimuli that refuse to fade away. Classical music has been shown to manifest 1/f-behavior [103,105] and to resonate with the human brain, leaving strains of melody running through your head long after the music stops. The influence of the more pleasant stimuli also resides on plateau III of Figure 14. West and Deering [123] reviewed the occurrence of the 1/f-variability in classical Western music, as well as the spatial variability in paintings by masters. They speculated that the aesthetic judgments we make regarding music and visual art may well have a biological origin in that the stimuli resonate with the complexity of the human brain. This once speculative postulate is now supported by experimentation, as well as being explained by a generalized LRT and the extension of the WH to the PCM [17].

As the networks in which we are immersed become increasingly complex, several apparently universal properties begin to emerge. One of these properties is the generalized version of the WH we expressed as the PCM in discussing the CCC. Another generalization has to do with how complex networks, perhaps involving phenomena from different STEM disciplines, exchange information with one another.

### 5.2. Memory and Generation Rate of CEs

The notion of having multiple types of memory usually disrupts the equanimity of even the most sanguine investigators accustomed to associating memory only with the length of time a given event influences subsequent events in a time series, typically as measured by the time it takes an auto-correlation function to decay to half its initial value. For example, an HRV time series is a manifestation of heartbeat dynamics and is determined by the joint action of two kinds of memory: one produced by unpredictable CEs (Crucial-memory type) and the other by Laplace determinism (Hamiltonian-memory type). Tuladhar et al. [108] emphasize that 1/f-variability has two distinct origins: one from each of these two independent memory sources. Moreover, they determined that meditation transforms the Hamiltonian-memory-type memory into a strongly coherent process while simultaneously transforming the Crucial-memory type from a condition of ideal 1/f-noise (μ=2) to a Gauss basin of attraction (μ=3). But we will have more on the influence of meditation on memory later.

The Hamiltonian-memory type includes memory resulting from FBM which can be derived from Hamiltonian dynamics that determines a network’s memory from the asymptotic vanishing of the auto-correlation function of a variable of interest. It also includes Laplace determinism. On the other hand, the Crucial-memory type is a consequence of the nonintegrable auto-correlation function generated by CE fluctuations. The two forms of memory can be distinguished using the correlation function and noting that in the case of CE infinite memory, i.e., the Crucial-memory type, the correlation vanishes after a single step, whereas in the case of FBM infinite memory, this is not the case: Both kinds of memory are hosted by HRV time series, and a balance between the two provides a measure of health.

A technique developed to discriminate between the two kinds of memory that actually distinguishes between CEs and pseudo-CEs is called renewal aging [124]. If the process being considered is a sequence consisting solely of CEs it is renewal, the probability of a CE occurring at time *t* is given by a convolution equation. If an event that occurs at time t=0 is observed at time $t^{\prime }>0$, then the nonstationary waiting time PDF is $\psi \left(t,t^{\prime }\right)$ with the corresponding nonstationary survival probability: $\Psi \left(t,t^{\prime }\right)=\overset{\infty }{\int_{t}}\psi \left(t^{\prime \prime },t^{\prime }\right)dt^{\prime \prime }$. The $t^{\prime }$-derivative of the survival probability simplifies to the form of a rate equation, and the time-dependent rate of generating CEs at time t is determined to have the asymptotic form [11]:

(16) $$R\left(t\right)=\left\{\begin{array}{c} \frac{1}{t^{2-\mu }}\;\;\mathrm{for}\;\mu <2\\ 1\;\;\;\;\;\;\mathrm{for}\;\;\mu >2 \end{array}\right..$$

Consequently, renewal aging in the nonstationary case with μ<2 is characterized by the number of CEs generated per unit time that decreases as an IPL in time, with an IPL index of 2−μ. In the stationary case with μ>2, the number of CEs generated per unit time is independent of time and does not fade.

### 5.3. MDEA Reveals Invisible CEs

We are interested in detecting invisible CEs, such those given by an experimental time series consisting of a mixture of CEs and pseudo-CEs where we can separate their influence. We performed this separation process using diffusion entropy analysis (DEA), which Scafetta and Grigolini [67] originally introduced to analyze CE time series. DEA enables an investigator to evaluate the correct scaling of a diffusion process. The PDF for the complex diffusion displacement variable X(t) has the following scaling form:

(17) $$p\left(x,t\right)=\frac{1}{t^{\delta }}F\left(\frac{x}{t^{\delta }}\right),$$

where δ is the scaling index, and the unknown PDF F(y) for CEs does not have the traditional Gaussian form. DEA measures the Wiener/Shannon (WS) entropy for the diffusion process such that inserting the scaling form of the PDF into the WS-entropy yields the following: $S\left(t\right)=A+\delta \log_{2}t$, where *A* is the constant reference entropy of the process whose properties are described by the unknown function *F*.

The diffusion entropy is seen to increase linearly on log-linear graph paper, with the logarithm of the time on the horizontal axis and the slope of the resulting straight line yielding the scaling coefficient δ. Other procedures to determine scaling such as detrended fluctuation analysis (DFA) are based on the scaling of the second moment, which can lead to a misinterpretation of the long-time behavior of the time series. The latter occurs when the PDF has an IPL tail that is sufficiently slow to generate a divergence. Before applying these ideas to empirical time series, let us use them to quantify the known properties of surrogate datasets to verify their utility.

We construct a surrogate dataset by converting a time series $\left\{\tau \right\}$ generated using Equation (8) into a random walk (RW) diffusive process by assuming that a random walker always jumps in the same direction by a given distance Δ at the sequential times $t_{1}=\tau_{1};\;$$t_{2}=\tau_{1}+\tau_{2}$; etc. This RW rule is established by setting ξ=0 when there are no events and ξ=1 when either a CE or a Poisson event (non-CE) occurs and the step size is taken to be Δ=1. The resulting surrogate time series ξ(t) is the superposition of non-CEs for μ>3 and CEs for μ<3:

(18) $$\xi \left(t\right)=\left(1-\epsilon \right)\xi_{\mu >3}\left(t\right)+\epsilon \xi_{\mu <3}\left(t\right).$$

The parameter ϵ<1 is the probability that the empirical signal is generated by a genuine self-organized temporal critical (SOTC) process [91]. In the case where the CEs generated by a SOTC are visible ϵ=1, the method of DEA leads to the detection of the proper scaling index δ. The waiting time IPL index is related to the scaling index by μ=1+1/δ when 3>μ>2; consequently, in this case, we have that δ=1/(μ−1).

**Figure 15:**
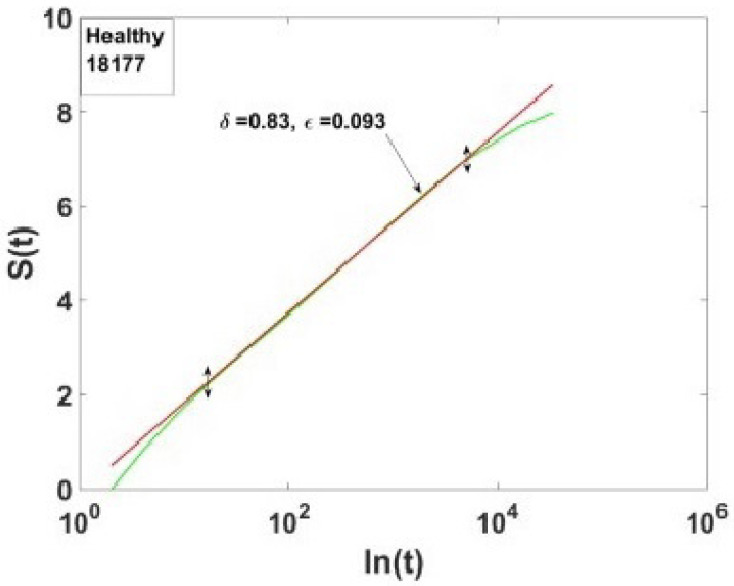
DEA detects the scaling of invisble CEs generated with ϵ=0.093 swamped in a sea of non-CEs in the intermediate asymptotic time. The solid green line is obtained for an empirical heartbeat dataset of a healthy individual. The scaling index δ=0.83 is the slope of the straight line between the two vertical arrows. From [91] with permission.

Figure 15 depicts the results of applying DEA to the above surrogate sequence with CEs embedded in a dense cloud of non-CEs generated by Mandelbrot’s FBM. These invisible CEs are detected in the intermediate asymptotic time domain using the DEA, but even more importantly, we have also included the results from a real heartbeat dataset for a healthy individual, as depicted by the green curve in the figure over nearly three orders of magnitude.

The scaling index δ is evaluated for the heartbeat dataset by monitoring the intermediate asymptotic region in the S(t) versus ln(t) graph through the time region between the vertical arrows, just as done for the surrogate data. In general, the time series generated by complex processes are characterized by three regimes: a short-time regime, where the true complexity of the process is not yet perceived; an intermediate-time regime driven by the CEs; and a long-time regime, where the process can be mistaken for an ordinary statistical process. The very-long-time regime is, on the contrary, a tempering effect generated by the self-organizing dynamic process itself.

Note that the procedure introduced here for revealing CEs is not sufficiently accurate to detect only renewal events. The events revealed by this analysis are a mixture of CEs and ordinary Poisson events. However, the presence of Poisson events does not prevent the detection of the anomalous scaling generated by CEs. The desired scaling was detected following Grigolini et al. [125], who generates a diffusion process X(t) using the rule that the random walker jumps ahead when either a CE or non-CE occurs. The scaling generated by Poisson events has a power law index of δ=0.5, whereas the scaling IPL index of CEs is given by the relation between scaling indices δ=1/(μ−1). Note that the latter scaling dominates asymptotically in time resulting in δ>0.5 when the condition 2<μ<3 applies [125]. The empirical dataset was used to generate the fluctuations in ξ(t) holding the value 1 when an event occurs, either a Poisson or CE, and a zero value when no event occurs. A moving window of size *t* then generates an ensemble of trajectories from which a histogram produces a scaling PD. This empirical PDF was then used to construct the WS information entropy as depicted in Figure 15, where the green curve is determined by the DEA of a diffusion process generated from empirical HRV datasets.

### 5.4. Principle of Complexity Synchronization (CS)

The last of the principles to be discovered was made by members of the *Center for Nonlinear Science* research group led by Grigolini and West [2,51]. In this recent series of papers, we have shown how to use the scaling behavior of empirical time series generated by the heart, lungs, and brain to hypothesize the existence of a new form of synchronization, *complexity synchronization* (CS), having to do with the optimally efficient exchange of information among the ONs within the human body viewed as the ultimate NoONs. CS is determined by the matching the MFDs of time series produced by interacting ONs. The phenomenon of CS was identified by processing 64-channel EEGs of human brains with each channel treated as a dynamic network with a unique scaling index, while the brain interacts with the all the physiological networks within the body and in particular with the H&L ONs. Consequently, using MDEA, we [1,2,51] processed 66 simultaneously recorded time series determining that the scaling index for every ON was in synchrony with every other ON, see the bottom panel of Figure 2. We cannot stress enough how remarkable we found that result to be.

Nature has apparently selected an optimally efficient way to handle information within the brain and its transmission to ONs elsewhere within the human body, as determined in a recent series of papers by West et al. [2] and Mahmoodi et al. [1,51]. The GW research group has used the scaling behavior of MFD time series to hypothesize the existence of a new form of synchronization giving rise to efficient exchange of information among the ONs within the human body’s NoONs. The fractal time series for channel-*j* of the EEG is given by $X_{j}\left(t\right)$, which satisfies a homogeneous scaling relation $X_{j}\left(\lambda t\right)=\lambda^{\delta_{j}\left(t\right)}X_{j}\left(t\right)$ determined by the scaling PDFs [11]: $P_{j}\left(x,t\right)=1/t^{\delta_{j}\left(t\right)}F_{j}\left(x/t^{\delta_{j}\left(t\right)}\right)$ (see SM1 for details).

The basis for the empirical CS formulated here is depicted in Figure 2, where 4 or 5 s of simultaneously measured time series are placed next to the appropriate ${\mathrm{ON}}_{j}$ drawing, and the bi-directional interactions among the triad of different ONs are indicated by arrows. The raw time series for the three kinds of ONs certainly do not appear to have anything in common. Yet, when the data were processed using the MDEA (see SM3), their scaling statistics were revealed to produce the MFD for the ${\mathrm{ONTS}}_{j}$ ($D_{j}\left(t\right)$) related to their IPL scaling indices ($\delta_{j}\left(t\right)$): $D_{j}\left(t\right)=2-\delta_{j}\left(t\right),\;$j=1,2,..,66. This is ostensibly the "bottom-up" role of synchrony in the behavior of NoONs, which is to say that the brain receives information from many if not all the ONs of the body from the five senses when excited by the environment and continuously from the ONs preforming necessary functions to keep the NoONs alive and healthy. The brain processes this information and selectively delivers the appropriate signals to the heart and lungs as well as to other ONs, thereby providing these two and the other "fractal machines" with suitably tuned operating instructions amidst an array of other tasks [1,2,51].

In general, the index for the scaling PDFs is a function of time $\delta_{j}\left(t\right)$ signifying that the empirical time series is multifractal, as indicated by the processed empirical 64-channel EEG datasets as well as those simultaneously measured from the heart and lungs depicted in the lower panel of Figure 2. Note that the time-dependent scaling index $\delta_{j}\left(t\right)$ is a direct measure of the complexity of the $j^{th}$ time series, thereby determining the information content of empirical time series, whatever the source [1,2,97]. The figure shows multifractal time series from each of the 66 ONs having the HBL ON-triad scaling indices in ’synchrony’ forming a quasi-periodic set of signals that we dubbed complexity synchronization (CS).

The quasi-periodic nature of the scaling parameter depicted in Figure 2 provides insight into the ways the information in the HBL ON-triad is exchanged among ONs during their mutual interactions. In this figure is depicted the instantaneous scaling index over all 64 EEG channels of the brain (gray curves), which is compared with the scaling index for the heart (blue curve), the scaling index of the lungs (pink curve), and the ‘scaling index for the brain’ obtained by averaging over the 64 channels of the EEG (black curve). This figure indicates that all the ONs (or 66 network channels) have dramatic changes in complexity over time, being a direct consequence of their inter-ON and intra-ON interactions. This time dependence of the scaling indices reflects the fact that the fractal dimensions of the ONTS become multifractal dimensions with quasi-periodic time dependencies.

The visual impression of the CS of the processed datasets in this figure is supported by the cross-correlation coefficients of the HBL-MFD scaling indices recorded [2] to reside in the narrow interval for the three pairs of cross-correlation coefficients [0.70, 0.73]. This synchronization of the multifractal behavior of each of the HBL-scaling indices is a clear manifestation of the CS phenomenon, which is not a strict deterministic mechanism but is a statistical regularity.

## 6. Discussion

Let us begin our discussion by providing concrete examples of how the proposed methods can improve clinical decision making or enhance existing techniques for signal analysis.

**Multifractal signal analysis (MFSA)**—which involves simultaneously measuring the scaling index δ(t) for different PTS (see Figure 2)—is a novel approach for objectively quantifying the real-time variable dynamics of inter-organ communication [1,2]. This is a new and superior paradigm to investigate the mathematical properties of Multiple Organ Dysfunction Syndrome (MODS), a clinical syndrome that is the result of a systematic breakdown in inter-organ communication: when four or more organs become functionally decoupled from the patient’s physiology, the chance for survival plummets to 0 % [126,127].

MFSA (Figure 2) has the *potential* to generate insight with improving clinical decision making in MODS; however, it still requires validation in a future set of empirical studies. It has **not yet** been applied to interpret physiological data explicitly in the context of MODS; the following is a testable hypothesis for its benefit in these kinds of patients based upon existing research [1,2,46]:

**Hypothesis 1.**  *Multifractal signal analyss (MFSA) can improve clinical decision making and treatments.*

Examples of how this hypothesis could be tested include the following:

**Improving triage**: It is not uncommon following trauma and acute injury for patients to have clinically silent progression of their injury prior to overt clinical deterioration. If we can identify and quantify the extent of injury progression while it is in its silent phase, then this may guide triage decisions so that these patients receive intervention before they enter the zone of irreversible clinical deterioration.

MFSA can be used to potentially identify disruption in inter-ON information exchange, which we hypothesize will be the harbinger for eventual clinical deterioration. The exact form that the scaling index δ(t) for a respective ON’s time series will take in the setting of irreversible acute deterioration remains unknown; one goal of future studies will be to explicitly make this known.

**Improving treatment:** Per the MFSA paradigm, every ON has a δ(t) profile corresponding to its healthy function in the setting of inter-ON communication [46]. Health in this context is the ability to be optimally physiologically adaptive in the setting of stress and environmental uncertainty [128].

Technology which measures, interprets, and communicates δ(t) in real-time to clinical decision makers can potentially help individualize intervention decisions for patients who are suffering from diseases of inter-ON communication. We propose to focus on MODS, as that both represents a clinically unmet need and will be mathematically interesting as the most extreme example of total inter-ON communication disruption.

However, there are several other ailments that arise from inter-ON communication disruption: demonstrating proof of utility of MFSA for MODS can set the precedent for motivating its use as a tool to probe other diseases.

**Improving design of life support devices:** Per the MFSA paradigm, life support devices (e.g., mechanical ventilators) should strive to recapitulate the healthy MFD scaling index δ(t) profile of the ON that is temporarily replacing [46,70]. This is because reproducing the healthy δ(t) can potentially correspond to optimal rehabilitation to healthy ON function and its dynamics to healthy inter-ON communication.

Our position in this matter based on existing evidence is that early studies with mechanical ventilators that replicated the natural respiration showed clinical promise because they were replicating the healthy δ(t) of normal respiration [53,129,130,131,132,133,134,135]. We aim to revisit the design of invasive mechanical ventilators—and other life support equipment—explicitly from the perspective of MSFA as it exists now, as we suspect that this holds promise for improving the state of the art in emergency ON replacement technology.

The most extreme example of systematic breakdown in inter-ON communication is MODS: we believe explicitly analyzing this disease process through our paradigm of MFSA will generate novel insight into the mathematical structure of total physiological disruption. The knowledge gained in this way has the potential to guide the design of future technology in life support equipment from a new set of mathematical first principles, where the design goal is to both replicate the function of lost ONs and replicate the ON’s communication with other ONs that comprise the patient’s physiology.

Finally, this knowledge will go a long way toward establishing a new paradigm for conceptualizing and interpreting physiological dynamics in health and disease, which can inform future health applications that seek to leverage physiological data to improve clinical outcomes. Preliminary conclusions have been drawn in this regard based on the data processing already done [46].

## 7. Conclusions

We conclude that the fractal nature of CETS suggests the lack of a single frequency or scale dominating the dynamics of any physiologic process. Therefore, holistic theories and methods invoking multifractal dimensionality of vastly different neurophysiological and behavioral processes interacting in nonlinear dynamic ways offer new and promising alternatives for better understanding communication among ONs within NoONs and among NoONs. Moreover, the matching among the various MFD time series within the human body supports the argument given by von Neumann for the existence of two distinct languages to support the operation of the mamallian brain.

Figure 2 provides a clear answer to the question: How does CS occur in scaled metrics from empirical datasets of HBL-triad ONs? For each of the 64 different EEG channels (gray lines), using stripes of a proper size made it possible to find the scaling index $\delta_{j},\;$j=1,2,⋯,64 in a sufficiently small bin of time Δt to define an ‘instantaneous’ value of $\delta_{j}\left(t\right),\;$j=1,2,⋯,64. This in itself is a significant benefit of using MDEA. This same method of analysis was applied to the lungs (red curve) and heart (blue curve) time series. While the interaction between the brain and the physiology ONs of the body has a number of conjectured forms in the physiology literature, Figure 2 and the attendent intrpretation of the MDFS by means of Figure 6 firmly establish that the complexity of these different physiological processes as measured by their respective MFD time series remain synchronized in the sence of CS.

A number of strong results have been presented in this tutorial; each in its own way is connected to the properties of complexity, CETS, and MFDS. The transdisciplinary nature of science as a whole became evident as the principles necessary to explain foundational issues in the physical, social, and life sciences blossomed into complexity theory and most recently to the discovery of CS. The science motif CS is based on scaling arising from the 1/f-variability of ONs and the need for an NoON to exchange information internally during intra-ON dynamics and externally during inter-ON dynamics. The working measure of complexity adopted herein is the MFD of CETS generated by an ON, and the difference in the MFDTS of two organ networks, which quantifies the relative complexity between interacting ONs.

The authors in [1,2,51] have established that information flows from ONs at a higher level of complexity to those at lower levels of complexity, as summarized in the ‘complexity matching effect’ (CME), and the flow is maximally efficient when the complexities are equal. Furthermore, they used the scaling of empirical time series from the HBL-triad of ONs to support the hypothesis that CS occurs between scaling indices or equivalently with the matching of the time dependencies among the ON’s MFDs within a NoONs.

Before listing the individual results discussed, we observe that analytic mechanics is concerned with reversible processes, whereas thermodynamics is concerned with irreversible processes, and there is no fundamental physical theory that spans the gap between the two; consequently, thermodynamics remains a heuristic theory. So our starting point was necessarily the working definition of complexity being given by the MFDs of the CETS. The theory for the information transfer among ONs and within a NoONs is consequenly heuristic, as is thermodynamics. Given this caveat, we draw the following conclusions:

(1) CEs are manifestations of cooperative interactions between the units of an ON that lead to a spontaneous self-organizing process, and for the life-sustaining networks considered herein, these are spontaeously generated by SOTC [16].
(2) CETS are a renewal process in which the time interval between successive CEs are statistically independent and may be dressed, with filling non-CEs in the intervals between CEs, or they may be bare, with no events filling those time intervals [11,95].
(3) The CCC shows that the demise of LRT only occurs asymptotically in a restricted domain of an ergodic ON stimulating a nonergodic ON.
(4) The CCC shows that the WH is valid asymptotically in a restricted domain of a nonergodic ON stimulating a responding ergodic ON.
(5) Information transfer between ONs is quantified using CETS as explained using the CCC to define the PCM&M. Multiple examples are drawn from complex interacting networks in the physical, social, and life sciences [11,17].
(6) The PCM&M has been empirically verified using MDEA to generate $\delta \left(t\right)$ and the MFDS to generate ϵ, which is the probability that an event is crucial, thereby locating an individual on the ($\delta ,\epsilon^{2}$)-plane. This method partitions healthy and pathological subjects in this parameter space by applying the insights gained from the CCC to empirical ECG time series [62,91].
(7) The MFD spectrum for healthy patients is broader than those with an illness or injury [17].
(8) The more complex a network, the broader its MFD spectrum, and the more information it contains [17]. Consequently, in conformity with the PCM&M, information is transferred from the network with the broader to that with the narrower MFD PSD [11].
(9) A promising tool for making further progress in the field of Network Medicine [136] was made by establishing the clear connection between MFD spectra and CETS [109], thereby suggesting a mathematical infrastructure for measures of CS.
(10) The new form of synchronization which we dubbed CS and which has been empirically determined [1,2,51] could just as easily have been called ‘multifractal dimension synchronization’ (MFDS) after the measure of complexity which manifests synchronization. It also suggests that if an ON is found having a different measure of complexity, we would expect the new measure to synchronize in accordance with the principle that optimizes the information exchange during an interacton.

### Key Principle: Information Flow Is Physical and Measurable

A fundamental principle of nature is that energy gradients drive the behavior of a physical system. A unique property of multiscale systems that are comprised of nested ONs is that information gradients can also drive physical system behavior [11,46]. This is entailed by the fact that multiscale systems have memory: that includes temporal memory—where the far past can influence the immediate future—and spatial memory—where processes that are far away (with respect to scale) can influence a local process.

Consequentially, energy flow is no longer the sole candidate to drive system behavior. Information gradients must be explicitly considered as well.

We augment our signal processing paradigm for PTS with this additional set of considerations:

- Assme all PTS are generated by a system that has multiscale memory until explicitly proven otherwise.
- This reinforces the role that non-Gaussian processes, which is to say CEs with IPL PDFs, play in generating PTS.

## Supplementary Materials

The following supporting information can be downloaded at: https://www.mdpi.com/article/10.3390/e27030241/s1.

## Nomenclature

The following abbreviations are used in this manuscript:

AFBM	aging FBM
BRV	breath rate variability
CAN	cardiac autonomic neuorpathy
CE	crucial event
CETS	CE time series
CCC	cross-correlation cube
CME	complexity matching effect
CS	complexity synchronization
CSH	CS hypothesis
DEA	diffusion entropy analysis
EEG	electroencephlogram
ECG	electracaediogram
FA	fractal architecture
FAH	FA hypothesis
FBM	fractional Brownian motion
FDE	fractional diffusion equaton
FFPE	fractional Fokker–Planck equation
FKE	fractional kinetic equaition
FRW	fractal RW
FOC	fractional-order calculus
FOPC	FO probability calculus
HBL	heart, brain, lungs
HRV	heartrate variability
HTD	heavy-tailed distribution
IF	information force
IOC	integer-order calculus
IOPC	IO probability calclus
IPL	inverse power law
lT	Information Theory
LRT	linear response theory
MFD	multifractal dimension
MFDTS	MFD time sries
MFDS	MFD synchronization
MDEA	modified DEA
MFSA	multifractal signal analysis
MLF	Mittag–Leffler function
MODS	Multiple Organ Dysfunction Syndrome
MSE	multiscale entropy
NoONs	network of ONs
ON	organ network
PCM	principle of compexity matching
PCM&M	PCM and management
PFA	principle of FA
PMFDS	principle of MFDS
PD	Parkinson’s disease
PDF	probability density function
PSD	power spectral density
PTS	physiological time series
RE	renewal event
RG	renormalization group
RW	random walk
SCPG	super central pattern generator
SHM	statistical habituation model
SFLE	simplest fractional Langevin equation
SM	supplementary material
SOC	self-organized criticality
SOTC	self-organized temporal criticality
SRV	stride rate variabilty
STEM	science technology engineering mathematics
WG	West/Grigolini
WH	Wiener hypothesis
WS	Wiener/Shannon
