# Supplementary material for: Principles Entailed by Complexity, Crucial Events, and Multifractal Dimensionality
Source: Entropy (Basel). 2025 Feb 26;27(3):241. doi: 10.3390/e27030241 (PMC11941117; doi:10.3390/e27030241)
Supplement: Supplementary file 1 [file entropy-27-00241-s001.zip › entropy-3425579-SM.pdf]

Supplemental Material for: "Principles Entailed by Complexity, Crucil Events and Multifractal Dimensinality"

Bruce J West and Senthil Mudaliar

1 SM 1: On CE time series & FOC

We hypothesized [18] that the ONs making up a NoON such as the human body each generates time series $X(t)$ consisting of a sequence of CEs modulated by a mulifractal dimension (MFD) index $\delta(t)$ such that scaling the time we obtain the scaling relation $X(\lambda t) = \lambda^{\delta(t)} X(t)$. Empirically the phenomenon of having a CE sequence is based on time series consisting of random discrete events with renewal statistics which enable two interacting organ-networks (ONs) to detect and quantify the complexity of the other ON and to minimize the difference in their relative complexity so as to become synchronized. The working quantification of complexity is given by the MFD which becomes equal in each ON time series (ONTS) even though they may be operating on largely different time scales, such as the brain, heart and lungs, see [7, 8, 19] for a discusision of the synchronixaion of the time-depenent scaling index $\delta(t)$. The discrete events in such time series have been named crucial because they emerge from the nonlinear complex sub-dynamics which determine the efficiency of information exchange among the members of the NoONs.

A crucal event time series (CETS) is generated by a renewal event time series (RETS) with an IPL waiting-time PDF $\psi(\tau) \propto \tau^{-\mu}$ having an IPL index in the domain $1 < \mu < 3$. Asymptotically, the generated CETS describes an ergodic process for the IPL index μ in the range $2 < \mu < 3$ with a finite average waiting time. Ergodic is the technical name for statistical processes for which time averages taken over time series are equal to those taken over PDFs. The understanding of complexity in many-body physics is formally understood by assuming the 'ergodic hypothesis' dating back over a century to the time of Boltzmann.

Zaslavsky et al. [10, 23] applied the renormalization group transformation R to the network dynamics such that the scaling properties of the incremental changes are: $R : \delta x = \lambda_X \delta x$; $R : \delta t = \lambda_T \delta t$, which apply, after some averaging, to a restricted space-time domain and the scaling parameters are $(\lambda_X; \lambda_T)$. They continue with the observation that the *fractal-order* kinetic

equation (*FOKE*) given by Eq.(1) is invariant under the renormalization group transformation:

$$R : D_t^{\alpha}\left[P\left(x,t\right)\right] \ = K_{\beta} R : D_{|x|}^{\beta}\left[P(x,t)\right] \tag{1}$$

which implies that the transformed fractal equation satisfies the scaling behavior:

$$\lambda_T^{\alpha} D_t^{\alpha}\left[P\left(x,t\right)\right] \ = K_{\beta} \lambda_X^{\beta} D_{|x|}^{\beta}\left[P(x,t)\right]. \tag{2}$$

The renomalization transformation may grneralized to obtain time-dependent scaling parameters $\alpha\left(t\right)$ and $\beta\left(t\right)$.

The lowest-order renormalization group solution is given by equating the renormalization parameters raised to their respective powers: $\lambda_T^{\alpha(t)} = \lambda_X^{\beta(t)}$. The solution to the renormalized *FOKE is* given in terms of the Fourier transform of the PDF, which is the characteristic function $\phi(k,t)$, expressed in terms of the Mittag-Leffler function $E_{\beta}(.)$ to be:

$$\phi\left(k,t\right) = E_{\beta}\left(-K_{\beta}\left|k\right|^{\beta} t^{\alpha}\right). \tag{3}$$

Consequently, taking the inverse Fourier transform of this characteristic function and expressing the Mittag-Leffler function as an infinte series, after some algebra [17, 23], results in the scaling solution for the PDF:

$$P\left(x\lambda_T^{\alpha/\beta}, \lambda_T t\right) = P\left(x,t\right)/\lambda_T^{\alpha/\beta}. \tag{4}$$

Selecting the ratio of the scaling parameters to be the exponent of the remaining scaling parameter: $\delta = \alpha/\beta$, and choosing the time scale of interest to reduce the dependence of the PDF on the left side of the equality in Eq.(4) to be dependent on the single scaled variable $x/t^{\delta(t)}$ using $\lambda_T = 1/t$, enables us to rewrite Eq.(4) in the form of the scaling PDF given by

$$P(x,t) = \frac{1}{t^{\delta(t)}} F\left(\frac{x}{t^{\delta(t)}}\right). \tag{5}$$

Thus, the solution to the *FOKE* yields the time-dependent scaling PDF along with the DEA data processing technique to interpret the datasets from the brain, heart and lungs in the text as CETS with synchronized levels of complexity as measured by their respective multifractal dimensions.

1.1 Solution to FOKE

The function $F(y)$ of the scaled variable $y = x/t^{\delta}$ in the scaled PDF given by Eq.(5) is unknown in general but is well known for certain values of the scaling parameter. Consider the time-independent case of he ordinary diffusion equation having the integer-order indices $\alpha = 1$ and $\beta = 2$ which yields the well-known scaling index $\delta = 1/2$ in which case the unknown function $F(y)$ becomes a Gaussian with the scaled variable $y = x/\sqrt{t}$. Note that the fractal dimension $\mathcal{D}$ for this process is obtained from $\mathcal{D} = 2 - \delta = 1.5$ which is therefore a monofractal process that is completely random, which is to say it is simple diffusion with no

memory. Consequently, scaling PDFs with $\delta \neq 1/2$ are anomolous in that their fractal dimension and $\mathcal{D} \neq 1.5$ and they contain various forms of memory.

If the anomoly has a completely temporal origin with $\alpha(t) < 1$ we have for the spatial derivative index $\beta = 2$ for a spatially homogeneous process and $\delta(t) < 1/2$ thereby producing the fractal dimension $\mathcal{D}(t) = 2 - \delta(t) > 1.5$ indicating the existence of a short-term memory process. The more the scaling index deviates from 1/2 the more the fractal dimension deviates from 1.5 and the stronger the anomoly. If the time deviation is produced by a fractal time series with an IPL waiting-time PDF which when these time intervals are statistically independent of one another constitues a *renewal* time series. The renewal time series is ergodic for $2 < \mu < 3$ with a finite average waiting time and is non-ergodic for $1 < \mu < 2$ with a diverging average waiting time.

Suppose the trajectory $X(t)$ crosses a known level at a specific time and we want to know how long we must wait to recross that same level. Given that the waiting-time PDF has the IPL form we denote the generic IPL index for the waiting-time PDF μ by the symbol $\mu_{\mathcal{S}}$. If however, the level of interest is the reset level $X(t) = 1$ the IPL indeex takes on a value characteristic of the CETS which we label $\mu_{\mathcal{D}}$. From the parameter relations in Table 1, it is clear that it is possible to prove that the IPL index is equal to the fractal dimension, so that we obtain [5, 19]:

$$\mu_{\mathcal{D}} = \mathcal{D} = 2 - \delta, \tag{6}$$

which is possible to establish using the probability of the diffusion trajectory returning to the origin which has been shown to be a renewal process and is consequenly a CETS.

The PSD $S_p(f)$ for a CETS is also IPL in terms of the frequency f:

$$S_p(f) \propto f^{-\beta}, \tag{7}$$

indicating $1/f-$variability and $\beta = 1$ yields 1/f-noise. This IPL PSD is the Fourier transform of a slowly decaying auto-correlation function indicative of a fractal time series having global or local self-similarity. The IPL indices μ and β are interrelated for the class of fractal time series of interest in medicine such that the fractal dimension given by Eq.(6) is related to the IPL PDF index defined by Eq.(7), and the IPL index for the PSD is [18]:

$$\beta(t) = 3 - \mu(t). \tag{8}$$

Thus, true $1/f-$noise at $\beta = 1$ arises only at the boundary $\mu = 2$ between the ergodic and non-ergodic domains.

The work of [1, 6] have demonstrated reduced multifractality associated with impaired parasympathetic control and pathological condition of congestive heart failure (CHF), respectively. This corresponds to a reduction of long-range correlations present in the HRV of healthy individuals, an outcome related to a loss of dynamical properties of the cardiovascular system. The control mechanism introduced in [21] is presented in terms of a fractional-order Focker-Panck equation (FOFPE) [11] which leads to investigations of HRV through

its PDF distribution. The FOFPE is a theoretical model that describes the evolution of the PDF of the physiologic process and its solution (when it can be obtained) captures the full dynamics of an ensemble of diffusion processes for CE time series [13].

2 SM 2: Stochastic Central Limit Theorem

The central limit theorem (CLT) determines the limit PDF of a statistical process to be Normal if the elements of the process satisfy a number of conditions. One such condition is that the second moment of the time series is finite. Paul Lévy proved in the 1920s that a stable limit PDF exists in the absence of this condition and the resulting stable PDF is named in his honor. In the same spirit a stochastic central limit theorem (SCLT) was devised to develop another extended CLT that is compatible with the FOC, specifically with the Mittag-Leffler function (MLF) solution to the Caputo FOC linear rate equation [9] and the PDF has the form:

$$E_\theta\left(-\left(\lambda t\right)^\theta\right)=\sum_{n=0}^{\infty}\frac{\left(-\left(\lambda t\right)^\theta\right)^n}{\Gamma\left(n\theta+1\right)}. \tag{9}$$

The SCLT is based on considering the limit of the probability P_S of detecting an event, which is to say, the probability that a CE is visible in an experiment. Each value of P_S generates a sequence of m elementary laminar regions but with only one visible CE at the end of the last laminar region. The SCLT focuses on the interval between two consecutive visible CEs and adopts a rescaling procedure to compensate for the incomplete-measurement induced survival probability enhancement [22].

Pramulkkul et al. [9] show that all waiting-time PDFs generating a non-integrable survival probability as a consequence of the SCLT yielding a PDF given by the negative of the time derivative of a Mittag-Leffler function (MLF). The proof leads to the conclusion that the survival probability given by a MLF $\Psi_{ML}(t)$ is universal in the same sense as the Normal and Lévy stable PDFs are universal each subject to a given set of constraints. We adopt the subscripts symbol to denote the MLF survival probability and the corresponding waiting-time PDF.

The concept of survival probability is here connected to the stochastic perspective of a complex network generating a CETS. The time interval between consecutive events (laminar region) which according to a coin tossing prescription are assigned the values +1 or -1. The initial network is prepared by selecting all the realizations with a CE occurring at that time, with ensuing positive laminar regions. Physical examples of the waiting-time PDF $\Psi_{ML}(t)$ generated by the cooperative interaction of many units are available [4, 22], with the important observation that the stretched exponential regime, which is the early time functional form of the MLF, becomes more extended as the probability of generating a visible CE decrease.

Assume that the time interval between consecutive CEs generated by a complex network under study is given by Eq.(9) with the IPL index in the domain $1 < \mu < 2$. The origin of this condition, usually interpreted as a manifestation of complexity, can either be the anomalous nature of the dynamics under investigation [2, 3] or the condition of criticality [14]. In the latter and less well-known situation the emergence of temporal complexity is due to the cooperative action of many interacting units as mentioned earlier. At the onset of the cooperation-induced phase transition from disorder to order, the mean field fluctuates, and its non-stationary waiting-time PDF corresponds to an IPL [15].

The proof of the SCLT was made for the imperfect detection of CEs and this is the reason we include it in this discussion. The case considered was of a detector used to monitor CEs having a probability of detecting these events of P_S that varies inversely with $\langle m \rangle$, and m is the random number of elementary time increments between two CEs. The condition $\langle m \rangle = 1/P_S \to \infty$ of the SCLT is quite different from the condition $m \to \infty$ of the Normal and Lévy CLTs, since m has very large fluctuations around $\langle m \rangle$ in the traditional arguments. The probability of generating a CE at time t that is the last of a sequence of m events does not satisfy the condition of generating a MLF stable form for $m \to \infty$. However, the waiting-time PDF of the time intervals between visible CEs, does generate a MLF stable form, for $P_S \to 0$.

The traditional CLT yields Normal distributions as the limit PDF. The generalized CLT developed by Lévy yields the α-stable distribution as the limit distribution. Finally, the SCLT presented by [9] yields the MLF as the limit distribution whose asymptotic form is:

$$\Psi_{ML}(t) = \frac{1}{\lambda \Gamma(\theta) t^{\theta}}, \tag{10}$$

so that the MLF distribution is asymptotically an IPL. Consequently, the corresponding waiting-time distribution is an IPL with index $\mu = 1 + \theta$ and consequently $0 < \theta < 1$.

3 SM 3: Modified Diffusion Entropy Analysis

Herein we present the main points of applying the MDEA to the processing of an empirical stochastic process to determine if such a time series has an IPL scaling index consistent with the theory presented in Secton 3 of the main text with additinal supporting discussion given in SM 1. To facilitate the material presented we provide a step-by-step construction of the MDEA beginning with an empirical ECG graph as an exemplar dataset from which we extract the discrete data points that makeup the CE time sereis. Note that this is not the typical HRV time series used in the cardiovascular literature which is given by the time intervals between successive heart beats.

The consistency between theory and empirical data is determined using the MDEA to process the simultaneously recorded times series generted by

the member of the HBL-triad of ONs. The main text in [7, 20] contains the results of applying this method to the 66 ONs of the triad. Each of the triad time series was processed following the seven sequential steps of the MDEA technique referenced to Figure 1:

1.) The first step is to project the data of each channel onto the interval [0,1] by normalizing each time series by the total time interval of the dataset. This enables the processing of each time series to be directly compared.

2.) The data profile over the unit interval is then divided into parallel stripes of size ΔT (Figure 1 a, ECG data).

3.) Inset in the figure displays what the events would look like when the stripe size is $\Delta T = 0.1$. The insert in the figure displays for visual clarity what the events would look like if the time resolution for the stripes was 0.1. Note that a slowly varying feature of the data trace would have a relatively few events well-spaced in time. However, a sharply peaked feature would have a large number of closely spaced events.

4.) The events are extracted by defining them as unit amplitude pulses if the signal at that time is in a different stripe with respect to its previous value (Figure 1b) and zero if it remains in the same stripe. Using the time series of the extracted events, we create a diffusion trajectory (Figure 1c), i.e., the cumulative sum of the events in Figure 1b.

5.) Determine the statistics of a single diffusion trajectory (blue curve in Figure 1c) by selecting a window size w and partitioning the diffusion trajectory into many pieces, each starting from an event.

6.) By initiating all the segments from an event, all the trajectories can be shifted to start from a common origin (Figure 1d) thereby forming the members of an ensemble.

7.) Finally, we evaluate the ensemble distribution of histograms at a given time (Figure 1e) because the events are statistically independent.

Following the above scheme the statistics of a single time-series diffusion trajectory correspond to that performed using the MDEA processing of the data. Start by picking a window size τ and slicing the empirical signal into many pieces, each of length $w = \tau$, each diffusion trajectory starting from an event (Figure 1d). This is accomplished by shifting all the slices to start from this origin n orde to evaluate the distribution (histogram) of trajectories at time τ (Figure 1e). Denoting the PDF for different window sizes as $P(x, \tau)$, we can define the SW-entropy as follows:

$$S(\tau) = -\int dx P(x, \tau) \log_2 P(x, \tau) \tag{11}$$

Assuming that $P(x, \tau)$ is the PDF corresponding to window size τ, we can define the diffusion entropy using the SW-entropy as being the average information

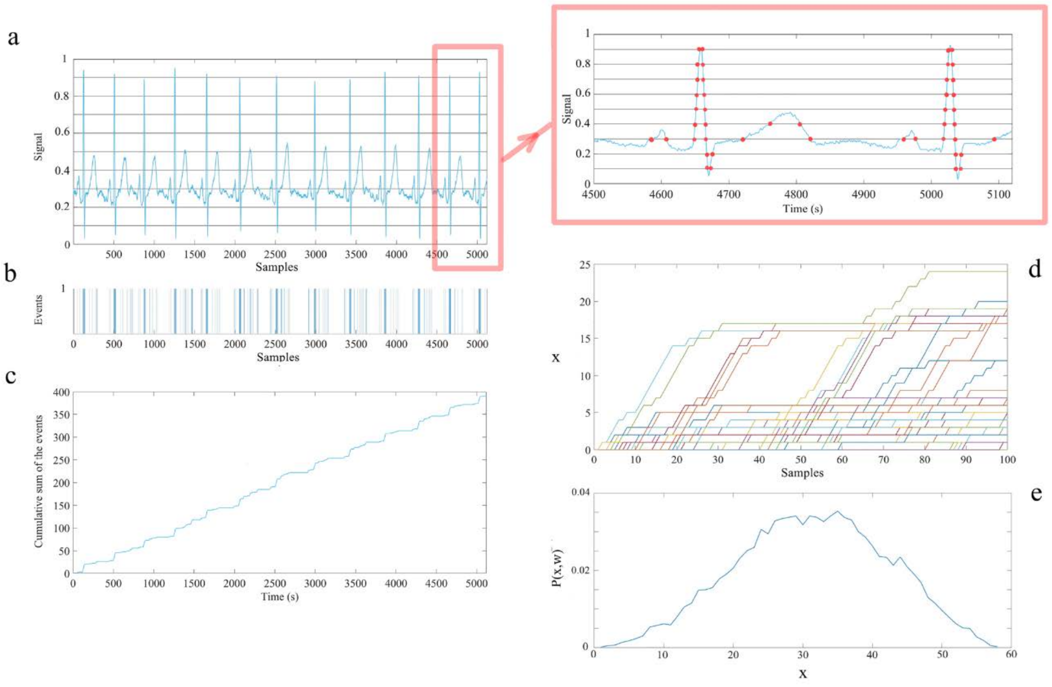

Figure S1:A schematic of the steps for the processing of time series using the technique of MDEA. Panel a): The solid curve is the heart rate signal projecte onto the intervsl [0,1] divided by the atripesmsize of 0.1 which is then magnified in the inset with the red border. Note that sharply praked features in the EG have clusters of events in panel (b), whereas a sloping featurehas well-spaced evens, see the inset for a visual verificationf this explnation. The horizontal lines define the stripes. Panel b): The events (represented as distinct separated unit positive amplitude pulses) are extracted from the passage of the continuous time trace of the ECG from one stripe to another. Panel c): The diffusion trajectroy made by the cumulative summation of the events of panel (b). The vertical lines show a selected set of windws with sizes 100 that sliced the diffusion trajectory. Panel d): The partitioned trajectory of panel (c) shifted to initiate each trajectory from a common origin and terminate each one after a time $w = t$, the length of the window. Panel e): The histogram of the position of the trajectories at the end of the windows (to crete this histogram 60 seconds od data and stripe width of 0.01,which were the DEA parameters used in the data processing.). Adapted from [7] with permission.

contained in the time series. Using the scaling PDF, without knowing the $F(.)$ function, the deviation of the SW-entropy from its reference state defined by the unknown function S_{ref} is:

$$\Delta S(\tau) \equiv S(\tau) - S_{ref} = \delta \ln \tau. \tag{12}$$

Consequently, if a graph of the SW-entropy for an empirical process versus the logarithm of the time τ yields a straight line with a positive slope, we can interpret that slope to be the scaling index δ.

This simple form for the growth in SW-entropy follows directly from the scaling form of PDF which enables us to deduce that the empirical time series has monofractal statistics. If the empirical slope of the time series is time dependent $\delta(\tau)$ it follows that the statstics are determined by a MFD process

Consequently, if a graph of the SW-entropy for an empirical process versus the logarithm of the time yields a straight line with a time-varying slope this is empirical evidence that the scaling index $\delta(\tau)$ yields the MFD time series for each of the 66 triads treated in the main text. Moreover it is emprical support of the theory.

References

[1] LA Amaral, et al. "Behavioral-independent features of complex heartbeat dynamics", *Phys. Rev. Lett.* **86**, 6026–6029 (2001).

[2] E. Barkai, Y. Garini, and R. Metzler, "Strange kinetics of single molecules in living cells," *Physics Today* **65**, 29 (2012).

[3] J. P. Bouchaud, "Weak ergodicity breaking and aging in disordered systems," *J. De Physique I,* vol. 2, no. 9, pp. 1705–1713 (1992).

[4] R. Failla, P. Grigolini, M. Ignaccolo, and A. Schwettmann, "Random growth of interfaces as a subordinated process," *Phys. Rev. E* **70**, Article ID 010101 (2004).

[5] FJ Feder. *Fractals*, Plenum Press: New York, NY (1988).

[6] PC Ivanov, et al. "Multifractality in human heartbeat dynamics", *Nature* **399**, 461–465 (1999).

[7] K Mahmoodi, SE Kerick, P Grigolini, PJ Franaszczuk, and BJ West, "Complexity synchronization: a measure of interaction between the brain, heart and lungs", *Sci. Rep.* **13**, 11433 (2023).

[8] K Mahmoodi, SE Kerick, P Grigolini, PJ Franaszczuk, and BJ West, "Temporal complexity measure of reaction time series: Operational versus event time", *Brain and Behavior* **13**(7): e3069 (2023).

[9] P. Pramukkul, A. Svenkeson, P. Grigolini, M. Bologna and B.J. West, "Complexity and the Fractional Calculus", *Adv. Math. Phys. Vol. 2013*, Article ID 498789, http://dx.doi.org/10.1155/2013/498789.

[10] AI Saichev and GM Zaslavsky, "Fractiomal kinetic equation: Solutions and applications", *Chaos* **7**, 753-764 (1997).

[11] I.M. Sokolov et al., "Linear response in complex systems: CTRW and the fractional Fokker–Planck equations", *Physica A* **302**, 268 (2001);

[12] RE Stake. *The art of case study research.* Sage Publications Ltd. London. (1995).

[13] *P Stein, et al.* "Sometimes higher heart rate variability is not better heart rate variability: results of graphical and nonlinear analyses". *J. Cardiovasc. Electrophysiol.* **16***,* 954–959 (2005). doi: 10.1111/j.1540-8167.2005.40788.

[14] E. Tagliazucchi, P. Balenzuela, D. Fraiman, and D.R. Chialvo, "Criticality in large-scale brain fMRI dynamics unveiled by a novel point process analysis," *frontiers in Physiology* **3**, article 15 (2012).

[15] M. Turalska, B. J. West, and P. Grigolini,."Temporal complexity of the order parameter at the phase transition," *Phys. Rev. E* **83**, Article ID 061142 (2011).

[16] BJ West, "A mathematics for medicine: the network effect". *Front. Physiol.| Fractal Physio.* **5**:456, (2014).

[17] BJ West, *Fractional Calculus View of Complexity: Tomorrow's Science*, see Section 7.6.1 Truncated Levy Process, CRC Press, (2016).

[18] BJ West and P. Grigolini, *Crucial Events: Why are Catastrophies Never Expected?,* World Scientific, Singapore (2021).

[19] BJ West, P Grigolini and M. Bologna, *Crucial Event Rehabiliation Therapy: Multifractal Medic*ine, Springer International Pub. (2023).

[20] BJ West, P Grigolini, SE Kerick, PJ Franaszczuk and K Mahmoodi, "Complexity Synchronization of Organ Networks"*, Entropy* **25**, 1393-1412 (2023).

[21] BJ West, "Fractal Calculus Facilitates Rethinking 'Hard Problems': A New Research Paradigm", *Fractal and Fract.***8**(11), 620-644 (2024)

[22] M. Zare and P. Grigolini, "Cooperation in neural systems: bridging complexity and periodicity," *Physical Review E* **86**, Article ID 051918, 6 pages (2012).

[23] GM Zaslavsky, "Chaos, fractional kinetics, and anomalus transport", *Phys. Rept.* **371,** 461 (2002).
